# Metabolic Reprogramming of Alloreactive T Cells Through TCR/MYC/mTORC1/E2F6 Signaling in aGvHD Patients

**DOI:** 10.3389/fimmu.2022.850177

**Published:** 2022-03-25

**Authors:** Zengkai Pan, Aijie Huang, Yang He, Zilu Zhang, Chuanhe Jiang, Luxiang Wang, Kai Qing, Sujiang Zhang, Jianmin Wang, Xiaoxia Hu

**Affiliations:** ^1^ Shanghai Institute of Hematology, State Key Laboratory of Medical Genomics, National Research Center for Translational Medicine at Shanghai, Ruijin Hospital, Shanghai Jiao Tong University School of Medicine, Shanghai, China; ^2^ Department of Hematology, Changhai Hospital, Shanghai, China; ^3^ Collaborative Innovation Center of Hematology, Shanghai Jiao Tong University School of Medicine, Shanghai, China

**Keywords:** allogeneic hematopoietic stem cell transplantation, metabolic reprogramming, T cells, aGvHD, glycolytic

## Abstract

Acute graft-versus-host disease (aGvHD) is the most common complication after allogeneic hematopoietic stem cell transplantation (allo-HSCT) and significantly linked with morbidity and mortality. Although much work has been engaged to investigate aGvHD pathogenesis, the understanding of alloreactive T-cell activation remains incomplete. To address this, we studied transcriptional activation of carbohydrate, nucleotide, tricarboxylic acid (TCA) cycle, and amino acid metabolism of T cells before aGvHD onset by mining the Gene Expression Omnibus (GEO) datasets. Glycolysis had the most extensive correlation with other activated metabolic sub-pathways. Through Pearson correlation analyses, we found that glycolytic activation was positively correlated with activated CD4 memory T-cell subset and T-cell proliferation and migration. T-cell receptor (TCR), mechanistic target of rapamycin complex 1 (mTORC1), myelocytomatosis oncogene (MYC) signaling pathways and E2F6 might be “master regulators” of glycolytic activity. aGvHD predictive model constructed by glycolytic genes (*PFKP*, *ENO3*, and *GAPDH*) through logistic regression showed high predictive and discriminative value. Furthermore, higher expressions of *PFKP*, *ENO3*, and *GAPDH* in alloreactive T cells were confirmed in our pre-aGvHD patient cohort. And the predictive value of the aGvHD risk model was also validated. In summary, our study demonstrated that glycolytic activation might play a pivotal function in alloreactive T-cell activation before aGvHD onset and would be the potential target for aGvHD therapy.

## Introduction

Allogeneic hematopoietic stem cell transplantation (allo-HSCT) is the solitary therapeutic modality for many malignant and non-malignant hematologic disorders. However, the benefits of allo-HSCT are challenged by graft-versus-host disease (GvHD), which affects >50% of patients and remains a major cause of mortality after allo-HSCT. The pathophysiology of acute GvHD (aGvHD) involves donor T cells and inflammatory cytokine-mediated injury to patient’s organ tissues (1). The Centre for International Blood and Marrow Transplant Research (CIBMTR) reported that within 100 days after allo-HSCT, aGvHD accounts for 13% and 16% of deaths in HLA-matched related and unrelated HSCT, respectively (2). The increased non-relapse mortality associated with GvHD may abrogate the favorable graft-versus-leukemia (GVL) effect on disease relapse.

The clinical outcomes of patients who suffered from grade III/IV aGvHD are dismal with a high mortality rate of 50%–70%, even with novel therapy including the Janus kinase (JAK)1/2 inhibitor and interleukin-2-inducible T-cell kinase inhibitors (3, 4). Numerous biomarkers have been identified to have a power to predict transplant-related mortality; however, they are the results caused by uncontrolled alloreactive T-cell activation (3, 5–7).

Metabolic regulation governs the fate and function of T cells, which plays a key role in aGvHD development. A growing body of evidence from multiple reports suggested that metabolic differences in immune cells may have significant association with GvHD pathology (8–10). However, few studies focused on the gene expression profile and metabolic shift in allo-activation of donor-derived T cells during aGvHD development. The influences of metabolic reprogramming and the gene–metabolite networks involved in aGvHD are not well characterized.

In the present study, through integration of T-cell gene expression signatures across different studies of aGvHD, we identified transcriptional activation of carbohydrate, nucleotide, tricarboxylic acid (TCA) cycle, and amino acid metabolism in alloreactive T cells before aGvHD onset. Glycolysis had the most extensive correlation with other enriched metabolic sub-pathways and might play a crucial function in alloreactive T-cell activation. Through Pearson correlation analyses, we found that glycolytic activation might contribute to the enrichment of activated CD4 memory T-cell subset and T-cell proliferation and migration. Glycolysis might be regulated by T-cell receptor (TCR), mTORC1, and MYC signaling pathways and transcription factor (TF) E2F6. High predictive value of the aGvHD risk model, which was constructed by glycolytic genes *PFKP*, *ENO3*, and *GAPDH*, further emphasizes the crucial function of glycolysis. Our study indicates that glycolytic activation might play a pivotal function in alloreactive T-cell activation for aGvHD development and would be a potential therapy of GvHD.

## Materials and Methods

### Data Collection and Preprocessing

Gene Expression Omnibus (GEO) (https://www.ncbi.nlm.nih.gov/geo/) provides an invaluable resource of high-throughput gene expression data that can be integrated and analyzed. Datasets from GEO that were considered eligible in our analysis met the following criteria: 1) Datasets with T-cell transcriptomic data from patients who developed aGvHD later (pre-aGvHD hereafter) and those who did not develop aGvHD (non-aGvHD hereafter); 2) Datasets with group information for each sample (pre-aGvHD vs. non-aGvHD); 3) Datasets with information about the technology and platform used for studies; and 4) Datasets with more than 10,000 probes. Based on the above criteria, two aGvHD datasets were downloaded from the GEO database. Details of each dataset are shown in **Table S1**.

### Identification of Differentially Expressed Genes

Bias of high-throughput experiments were commonly derived from heterogeneity and variables. In this study, we recruited datasets from different platforms and samples and handled by individual researchers. To avoid the possible unreliable results, samples from the two datasets were integrated to increase the number of samples (37 pre-aGvHD samples vs. 48 non-aGvHD samples), followed by batch normalization using R package “sva” (11). Next, gene differential analysis (|LogFC| >1, *P* < 0.05) was performed by comparing T cells isolated from pre-aGvHD and non-aGvHD blood samples using R package “limma”. Afterward, volcano map and heatmap were depicted by “limma” package and “pheatmap” package, respectively, in the R computing environment (12).

### Single-Sample Gene Set Variation Analysis

Gene Set Variation Analysis (GSVA) was applied to calculate the activities of metabolic and signaling pathways for each sample (13). Pathway signatures were obtained from MSigDB of the Broad Institute (https://www.gsea-msigdb.org/gsea/index.jsp) and Reactome annotation (https://reactome.org) (14, 15). For comparison of GSVA scores, the T-cell expression data were multiplied by 1 for aGvHD-dependent lines or by −1 for aGvHD-independent lines to reflect the direction of aGvHD dependency (positive for aGvHD dependency and negative for aGvHD independency). The data were then standardized to z-scores across samples for comparison and the creation of correlation matrix heatmaps.

### Pathway and Gene Correlation Analysis

The correlation of gene expression level and pathway score derived from GVSA was evaluated by Pearson correlation. |Correlation coefficient| >0.4 and *P* < 0.05 were considered statistically significant.

### Immune Cell Profiling Analysis

We used the CIBERSORT tools (16) to identify immune cell profile from the RNA expression data.

### Prediction of Transcription Factors

TFs potentially driving the expression of glycolytic genes and metabolic reprogramming were predicted by the module “UCSC_TFBS” under the “Protein_Interactions” function of DAVID (https://david.ncifcrf.gov/home.jsp) and Pearson correlation analysis. TFs regulating the expression of individual gene (such as *PFKP*, *ENO3*, or *GAPDH*) were predicted through R package “TFBSTools” (17) and JASPAR database (http://jaspar.genereg.net/). The target genes of E2F6 were predicted using hematopoietic cell-specific chromatin immunoprecipitation followed by sequencing (ChIP-seq) data collected in Cistrome data browser (18).

### Multivariable Logistic Regression Analysis

Glycolysis-associated genes, *PFKP*, *ENO3*, and *GAPDH*, were applied to develop an aGvHD risk model. Nomogram was constructed to predict aGvHD risk with multivariate logistic regression analysis. To assess the calibration of the risk model, calibration curves were plotted. To quantify the discrimination performance of the risk model, Harrell’s C-index was measured, and receiver operating characteristic (ROC) curve was drawn. Furthermore, the risk model was subjected to bootstrapping validation (1,000 bootstrap resamples) to calculate a relatively corrected C-index. To determine the clinical usefulness of the risk model, decision curve was plotted through calculating net benefits across different threshold probabilities.

### Single-Cell RNA Sequencing

CD3^+^ T cells were collected from 5 non-aGvHD patients and 9 pre-aGvHD patients by EasySep™ Human T Cell Enrichment Kit (Stemcell Technology, Cat # 19051). For 5′ single-cell RNA sequencing (RNA-seq) data, raw reads obtained from the 10× Genomics single-cell RNA-seq platform were demultiplexed and mapped to the human reference genome GRCh38 using the CellRanger software (version 3.0.2) (https://support.10xgenomics.com/single-cell-gene-expression/software) with default parameters. In this study, CD3^+^ cells were retained for downstream analysis. Finally, our study included 48,718 CD3^+^ T cells. Normalized gene expression values were plotted for each cell as violin plot in R.

### Flow Cytometry

For the analysis of cell surface molecules, single-cell suspensions were prepared. CD4^+^ memory T cells (CD4^+^CD45RO^+^) were detected by flow cytometry.

### Study Approval

The study was approved by the Ruijin Hospital Ethics Committee, and all processes were consistent with Helsinki Declaration standards.

### Statistical Analysis

The differentially expressed genes (DEGs) and GSVA results were displayed with *P* values, fold changes, and ranks. An unpaired two-tailed Student’s t-test (for two group comparisons) or a one-way ANOVA was performed, and the Wilcoxon rank-sum test was performed using R package ggplot2. The results of multivariate logistic regression were displayed with odds ratio (OR) and *P* values. The strength of the Pearson correlation was displayed with correlation coefficient as the following guide: 0.00–0.19, “very weak”; 0.20–0.39, “weak”; 0.40–0.59, “moderate”; 0.60–0.79, “strong”; 0.80–1.0, “very strong.” *P* value <0.05 was statistically significant. All statistical tests and graphing were performed by R. In the figures, statistical significance was shown as follows: *P < 0.05, **P < 0.01, ***P < 0.001, and ****P < 0.0001.

## Results

### Metabolic Reprogramming of Alloreactive T Cells in aGvHD Patients

**Figure 1:**
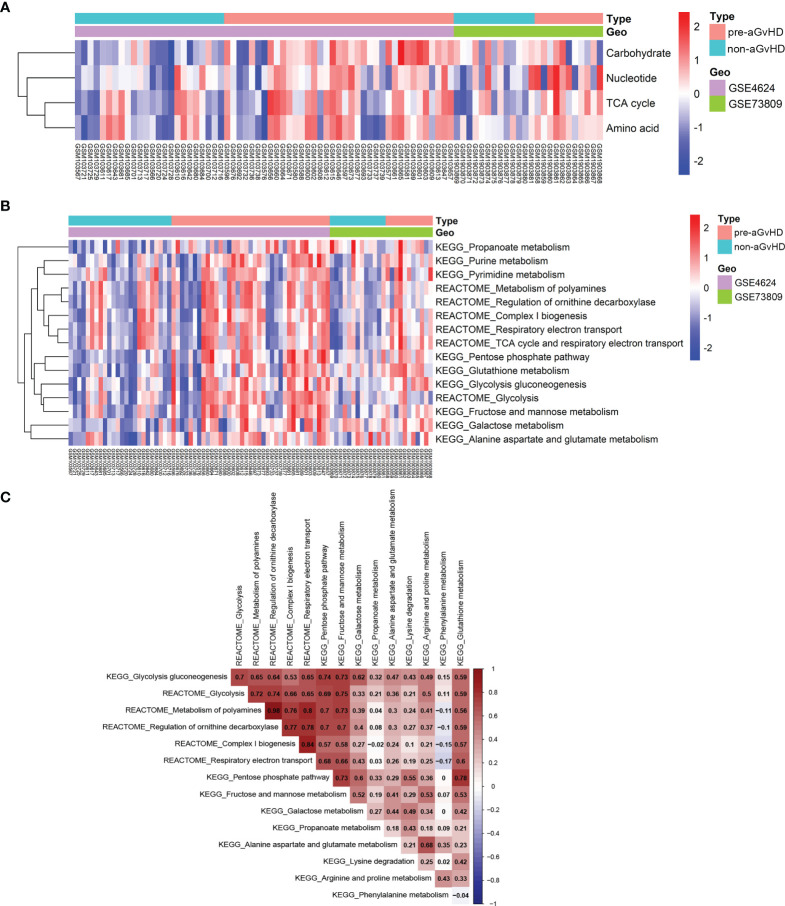
Distinct metabolic reprogramming based on pathway gene expression. **(A)** Heatmap shows enrichment of four super-metabolic signatures across each sample. The right longitudinal axis indicates the names of metabolic signatures. The left longitudinal axis represents clustering of metabolic signatures. Red denotes high activity and blue denotes low activity of metabolic pathways. Differentially enriched pathways with *P* < 0.05 and |log FC| >1 were considered significant. The terms indicated are as follows: Carbohydrate, carbohydrate signature; Nucleotide, nucleotide signature; TCA cycle, tricarboxylic acid (TCA) cycle signature; Amino acid, amino acid signature; pre-acute graft-versus-host disease (aGvHD), T-cell samples from patients who further developed acute graft-versus-host disease; non-aGvHD, T-cell samples from patients without graft-versus-host disease. **(B)** Heatmap shows different activities of metabolic sub-pathways derived from carbohydrate, nucleotide, TCA cycle, and amino acid signatures between pre-aGvHD and non-aGvHD samples. **(C)** Correlations of metabolic sub-pathway activities with each other. Color indicates the correlation direction. Each cell contains Pearson correlation coefficient.

To explore transcriptional metabolic reprogramming of T cells before full onset of aGvHD, we integrated the T-cell transcriptomic data across different studies (19, 20). The raw data and platform information of GSE4624 and GSE73809 were downloaded from GEO database. Both studies collected T cells prior to aGvHD onset (pre-aGvHD group) and on matched time in control patients who were without aGvHD (non-aGvHD group) and concluded that gene expression signature of alloreactive T cells had a dominant effect on the development of aGvHD. After annotation, 8,455 genes in GSE4624 (GPL3639) and 30,905 genes in GSE73809 (GPL17586) were obtained (**Table S1**). To reduce the possibility of false positive results, the two datasets were batch normalized and integrated (**Figure S1**). The expression patterns of metabolic pathway genes have been determined to reflect actual metabolic activities in patients well (14). To analyze the metabolic activities of each sample, we examined the gene sets of seven super-pathways based on Reactome annotation (14, 15) including amino acid metabolism (348 genes), carbohydrate metabolism (286 genes), energy metabolism (110 genes), lipid metabolism (766 genes), nucleotide metabolism (90 genes), TCA cycle (148 genes), and vitamin and cofactor metabolism (168 genes) with GSVA approach (**Table S2**) (13). Next, we compared the enrichment scores of seven metabolic signatures between T cells from pre-aGvHD and non-aGvHD samples. The results showed that carbohydrate, nucleotide, TCA cycle, and amino acid metabolism were significantly enriched in pre-aGvHD samples (**Figure 1A**, **Table S3**). Of note, carbohydrate metabolism was the most significantly enriched (*P* = 0.0003). To identify the metabolic pathways dysregulated in pre-aGvHD samples, we examined sub-metabolic gene sets of the four enriched super-pathways based on Reactome and Kyoto Encyclopedia of Genes and Genomes (KEGG) annotation. The sub-pathways with high activity in pre-aGvHD samples were mainly glycolysis-related, such as glycolysis, pentose phosphate pathway, fructose and mannose metabolism, galactose metabolism, and respiratory electron transport (**Figure 1B**), of which glycolysis had the most extensive correlation with other enriched sub-pathways **(** **Figure 1C**). These results suggested that glycolysis might be the crucial metabolic pathway. The extensively activated pathways might be the results of glycolysis activation before aGvHD full onset.

### Glycolytic Genes Differentially Expressed in Alloreactive T Cells Before aGvHD Onset

**Figure 2:**
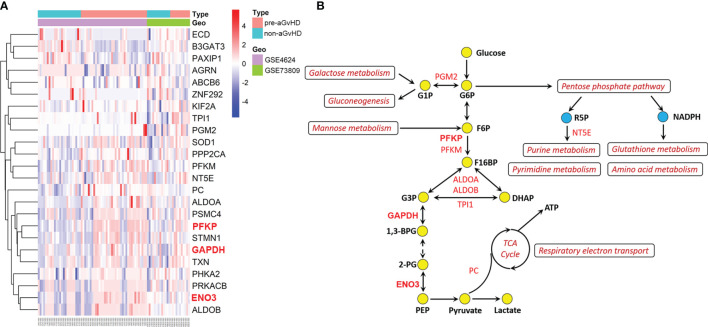
Dysregulation of glycolytic genes and glycolysis-associated metabolic pathways. **(A)** Heatmap and hierarchical clustering of differentially expressed glycolytic genes. The right longitudinal axis indicates names of differentially expressed genes. The left longitudinal axis represents clustering information. The red color represents the upregulated genes; the blue color represents the downregulated genes; the white color represents genes without change. Significance was defined in **Figure 1A**, above. **(B)** Schematic overview of upregulated glycolytic genes and activated glycolysis-associated metabolic pathways. Red text indicates upregulated genes, and dark red text indicates activated pathways. The yellow circles represent glycolytic metabolites, and the blue represents metabolites of pentose phosphate pathway. G1P, glucose-1-phosphate; G6P, glucose-6-phosphate; F6P, fructose 6-phosphate; F16BP, fructose 1,6-bisphosphate; G3P, glyceraldehyde 3-phosphate; DHAP, dihydroxyacetone phosphate; 1,3-BPG, 1,3-bisphosphoglycerate; 2-PG, 2-phosphoglycerate; PEP, phosphoenolpyruvate; TCA, tricarboxylic acid; ATP, adenosine triphosphate; R5P, ribose 5-phosphate; NADPH, nicotinamide adenine dinucleotide phosphate.

To further identify the genes involved in the activation of glycolysis employed by T cells in the pre-aGvHD state, we performed differential expression analyses of genes derived from REACTOME_Glycolysis and KEGG_Glycolysis_gluconeogenesis gene sets. Compared with T cells isolated from non-aGvHD samples, 18 glycolytic genes were upregulated and six genes were downregulated in pre-aGvHD group (|Fold Change| >1 and *P* < 0.05; **Figure 2A**, **Table S4**). The top 5 upregulated genes were *PFKP*, *SOD1*, *ENO3*, *GAPDH*, and *STMN1*. Among them, *PFKP*, *ENO3*, and *GAPDH* have well-known functions in glycolysis. Phosphofructokinase (encoded by *PFKP*) catalyzes the phosphorylation of fructose 6-phosphate (F6P) to fructose 1,6-bisphosphate (F16BP) by ATP, which is the most important control step of glycolysis (21). Enolase (encoded by *ENO3*), also known as phosphopyruvate dehydratase, catalyzes the transformation of 2-phosphoglycerate (2-PG) to phosphoenolpyruvate (PEP) (22). Glyceraldehyde-3-phosphate dehydrogenase (encoded by *GAPDH*) is a glycolytic enzyme that catalyzes the critical step by converting glyceraldehyde 3-phosphate (G3P) into 1,3-bisphosphoglycerate (1,3-BPG). Inhibition of glyceraldehyde-3-phosphate dehydrogenase (GAPDH) downregulates glycolysis in both myeloid and lymphoid cells, preventing immune activation (23–25). Participation of upregulated genes in glycolysis and their interaction with other metabolic pathways are visualized in **Figure 2B**.

### Metabolic Reprogramming and Glycolytic Genes Are Associated With T-Cell Activation Signature in aGvHD Patients

**Figure 3:**
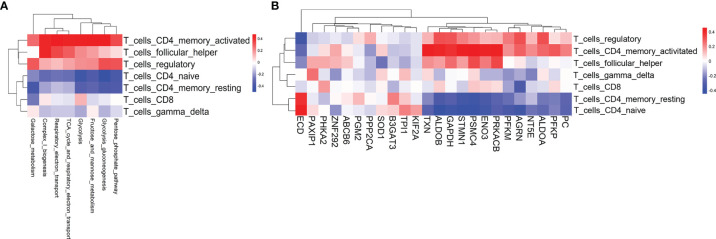
T-cell subsets associated with glycolysis-related pathways and glycolytic genes. **(A)** Heatmap of the Pearson correlation coefficients between glycolysis-related metabolic signatures and T-cell subset signatures. **(B)** Heatmap of the Pearson correlation coefficients between glycolytic genes and T-cell subset signatures. The information for the T-cell subsets was obtained using CIBERSORT.

In the pathogenesis of aGvHD, donor-derived alloreactive T cells are activated by recognition of host antigens, then adopt a pathogenic effector phenotype and migrate to target organs. Activated T cells require more energy to proliferate and mediate effector functions. However, the underlying relationship between metabolic reprogramming and alloreactive T-cell activation is still poorly understood (10). To assess the biological relevance of metabolic reprogramming, we evaluated T-cell subsets and T-cell functional signatures by GSVA. We first scored seven T-cell subsets for their relative abundance by using CIBERSORT (16) and evaluated the correlation of the T-cell subsets with metabolic reprogramming and differentially expressed glycolytic genes. Activated CD4 memory T cell was identified to be the top subset that positively correlated with glycolysis-related pathways and glycolytic genes (**Figures 3A, B** **)**. The expressions of *TXN*, *ALDOB*, *GAPDH*, *STMN1*, *PSMC4*, *ENO3*, *PRKACB*, and *PFKP* had strong correlations with activated CD4 memory T subset (**Figure 3B**). Furthermore, we validated the positive correlation between CD4 memory T subset with aGvHD development and glycolysis activation in our patient cohort. We found that the proportion of CD4 memory T cells was significantly higher in pre-aGvHD samples than their counterparts in non-aGvHD group (13.16% ± 6.585% vs. 1.378% ± 0.8093%, *P* < 0.05) (**Figure S2A**). Single-cell RNA sequencing data revealed significantly higher expression of glycolytic genes PFKP and GAPDH in CD4 memory T cells of pre-aGvHD patients than non-aGvHD patients (**Figure S2B**), supporting the important function of glycolytic activation in CD4 memory T cells during the aGvHD development. Next, we examined enrichment of T-cell functional signatures derived from gene ontology (GO) for validation. T-cell proliferation and migration signatures were positively associated with glycolysis-related pathways and glycolytic genes (**Figures S3A, B**). The expressions of *ALDOB*, *ENO3*, *PRKACB*, *STMN1*, *TXN*, *PFKP*, *GAPDH*, *ALDOA*, *PFKP*, *AGRN*, *PSMC4*, and *SOD1* had significant correlations with T-cell proliferation and migration (**Figure S3B**). Overall, these results suggested that metabolic activity was intrinsically coupled with T-cell activation pathways.

### Hallmark Signaling Pathways and Transcriptional Factors Involved in Metabolic Reprogramming and Differentially Expressed Glycolytic Genes

**Figure 4:**
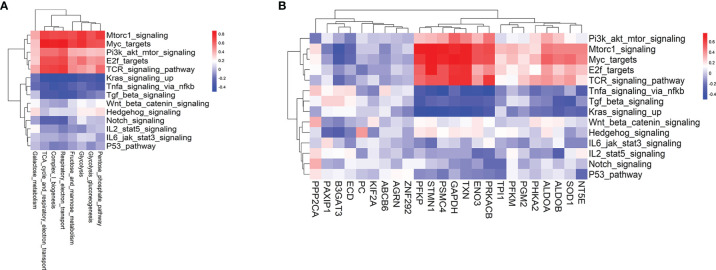
Hallmark signaling pathways associated with glycolysis-related pathways and glycolytic genes. **(A)** Heatmap of the Pearson correlation coefficients between glycolysis-related metabolic signatures and hallmark as well as T-cell receptor (TCR) signaling pathways. **(B)** Heatmap of the Pearson correlation coefficients between glycolytic genes and hallmark as well as TCR signaling pathways. The hallmark signaling and TCR signaling gene sets are based on MSigDB.

Metabolic reprogramming is largely determined by signaling pathways and TFs. TCR, the phosphatidylinositol 3-kinase (PI3K)/protein kinase B (AKT), mTOR, MYC signaling, and TFs such as IRF4, SREBP, PGC1α, HIF1α, ATF4, and E2F have been widely involved in anabolic or catabolic metabolism, including glycolysis and REDOX balance (14, 26–28). Thus, we compared the enrichment scores of hallmark signaling and TCR signaling pathways between T cells from pre-aGvHD and non-aGvHD samples. The results showed that MYC, mTORC1, TCR, Hedgehog, and Wnt/β-catenin signaling pathways were significantly enriched in pre-aGvHD groups (**Figure S4**, **Table S5**). To identify specific driver signaling pathways, we performed correlation analyses of metabolic reprogramming and differentially expressed glycolytic genes with hallmark signaling and TCR signaling pathways. TCR, mTORC1, and MYC signaling pathways were identified as drivers with top positive correlations (**Figures 4A, B** **)**. The expressions of *PFKP*, *STMN1*, *PSMC4*, *GAPDH*, *TXN*, *ENO3*, and *PRKACB* had stronger correlations (Correlation coefficients >0.5 and *P* < 0.05) with TCR, mTORC1, and MYC signaling activity (**Figure 4B**).

Gene expression is generally regulated by TFs directly, which consequently influences metabolic activity. To elucidate TFs that potentially drive the expression of glycolytic genes and metabolic reprogramming, DEGs between pre-aGvHD and non-aGvHD groups (**Figures S5, S6**) were utilized for the prediction of TFs by the module “UCSC_TFBS” under the “Protein_Interactions” function of DAVID (https://david.ncifcrf.gov/home.jsp). TFs predicted with adjusted *P* < 0.05 were considered to be significantly enriched (**Table S6**). The enriched TFs and other well-known metabolism-regulating TFs (26, 27, 29) whose expression data are available to access in this study (E2F1, E2F4, E2F6, MYC, ATF4, ATF6, NFKB1, STAT3, LPIN1, HIF1A, RPS6KB2, YY1, SREBF2) were submitted for the following analyses. We performed correlation analyses of metabolic reprogramming and differentially expressed glycolytic genes with the abovementioned TFs. E2F6 was identified as the top positively correlated TF (**Figures S7A, B**), which was consistent with the positive correlation of E2F target signature with metabolism reprogramming and glycolytic gene expression (**Figures 4A, B** **)**. Furthermore, expressions of MYC, MTOR, and E2F6 were proven to be higher in pre-aGvHD T cells than those in non-aGvHD T cells by our own patient cohort **(** **Figure S8A**). These results suggest that TCR, mTORC1, and MYC signaling pathways and E2F6 are potential “master regulators” of metabolic reprogramming and glycolytic activity.

### Development of an Individualized aGvHD Predictive Model

**Figure 5:**
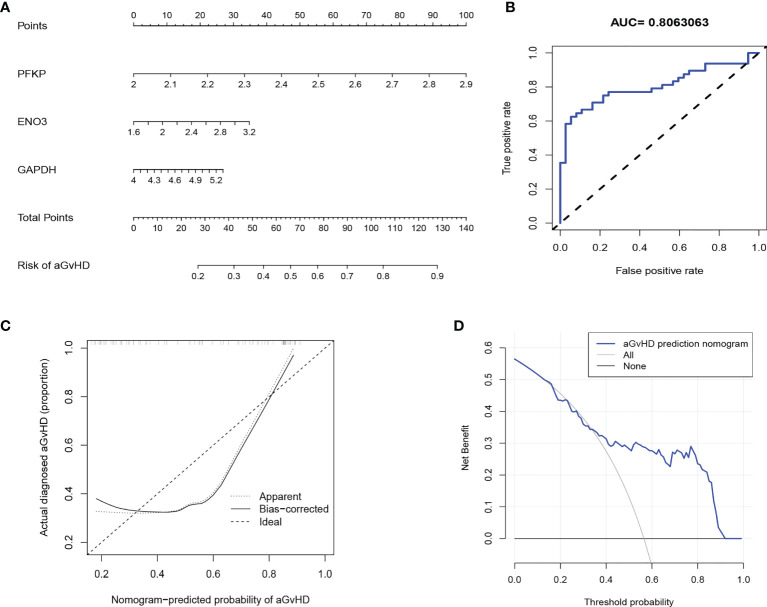
Development of an individualized predictive model for acute graft-versus-host disease (aGvHD) with logistic regression. **(A)** Nomogram of aGvHD prediction. The aGvHD nomogram consists of three glycolytic genes, namely, *PFKP*, *ENO3*, and *GAPDH*. **(B)** Receiver operating characteristic (ROC) curve shows the predictive value of the risk model. **(C)** Calibration curves of the aGvHD risk model. The x-axis represents the predicted aGvHD risk. The y-axis represents the actual diagnosed aGvHD. The diagonal dotted line represents the ideal predictive model. The solid line represents the performance of our risk model, of which a closer fit to the diagonal represents a better prediction. **(D)** Decision curve for the aGvHD risk model. The y-axis measures the net benefit. The blue line indicates the aGvHD risk model. The thin solid line represents the assumption that all patients develop aGvHD. The thick solid line represents the assumption that no patient develops aGvHD.

Regarding the central function of glycolysis in alloreactive T-cell activation in pre-aGvHD samples, we used *PFKP*, *ENO3*, and *GAPDH*, which were among the most significantly upregulated glycolytic genes and their expressions were proven to be higher in pre-aGvHD T cells than those in non-aGvHD T cells **(** **Figure S8B** **)**, to construct an aGvHD predictive model. The predictive model that incorporated the three predictors was developed and presented in the nomogram (**Figure 5A**). The area under the ROC curve was 0.806, showing a high predictive value (**Figure 5B**). The C-index of the nomogram was 0.806 (95% CI: 0.713–0.899) for the cohort and was confirmed to be 0.786 through bootstrapping validation, suggesting that the risk model had good discriminative ability. The calibration curve demonstrated that the nomogram had good concordance to predict aGvHD risk in this cohort (**Figure 5C**). The decision curve showed that the aGvHD predictive model provided superior net benefit when clinical decision thresholds were between 14% and 91% (**Figure 5D**). Furthermore, the predictive value of the aGvHD risk model was validated by our own patient cohort (**Figures S8C, D**).

**Figure 6:**
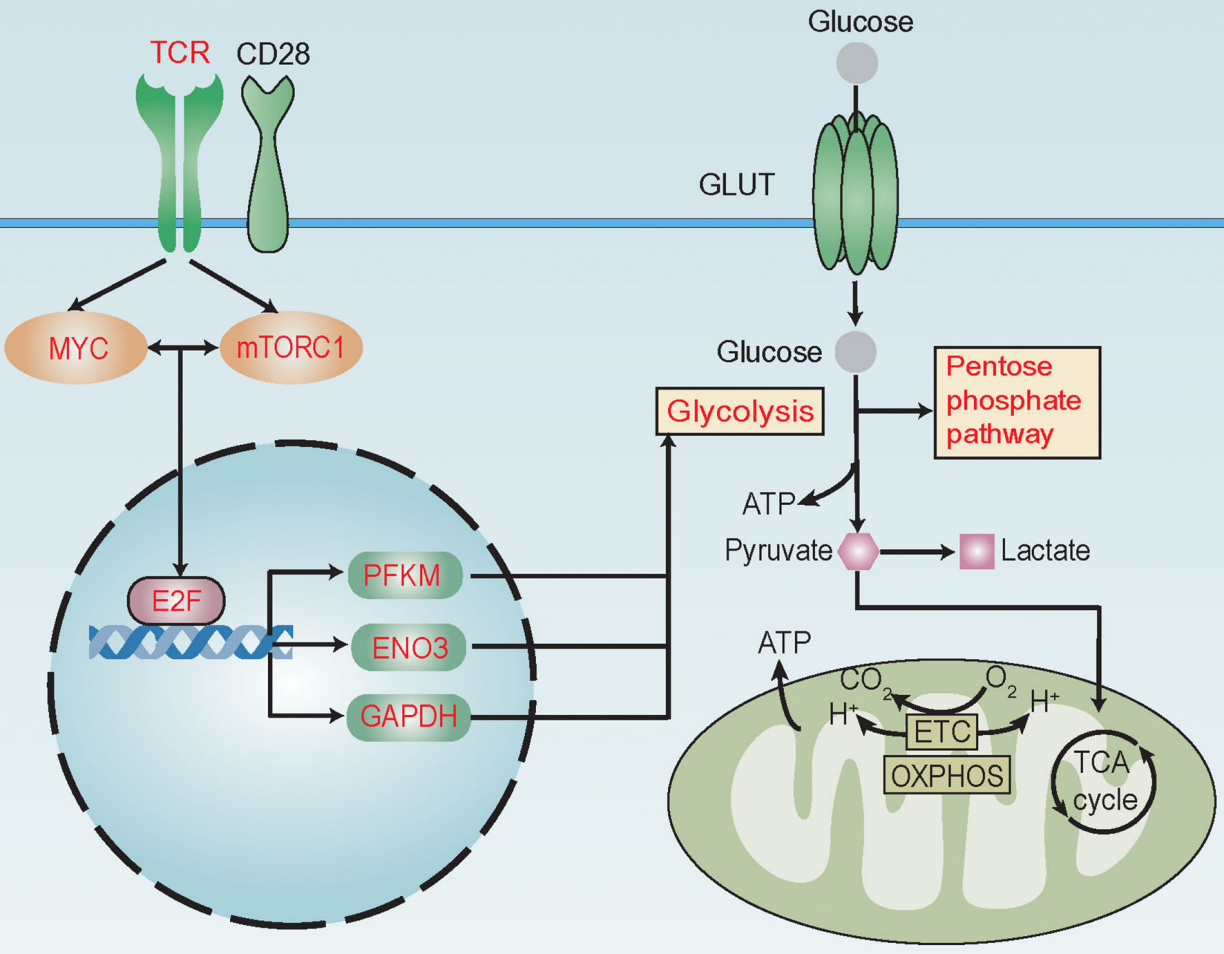
Reprogramming of glucose metabolism in T cells during acute graft-versus-host disease (aGvHD) pathogenesis. MYC and mTORC1 signaling pathways are known to be activated by T-cell receptor (TCR) and co-stimulating molecular CD28 during antigen recognition to promote the transcriptional activity of E2F family transcription factors (TFs). During aGvHD pathogenesis, we found glycolysis and its related pathways, such as pentose phosphate pathway, to be enriched in alloreactive T cells. TCR, mTORC1, MYC signaling pathways and E2F6 might be “master regulators” of glycolytic activity. Expressions of glycolytic genes, such as *PFKP*, *ENO3*, and *GAPDH* were shown to be elevated and correlated with activities of TCR, MYC, and mTORC1 signaling and E2F family. E2F6 was predicted as a TF to regulate the expression of *PFKP*, *ENO3*, and *GAPDH.* High expression of glycolytic genes *PFKP*, *ENO3*, and *GAPDH* in T cells can predict aGvHD development. Thus, we assumed that TCR might interact with MYC and mTORC1 to promote E2F expression, further increasing the expression of *PFKP*, *ENO3*, and *GAPDH* to activate glycolysis and its related pathways in alloreactive T cells during aGvHD development. These changes in glucose metabolism might promote T-cell activation through supplying energy and precursors for anabolism. Red text indicates activated signaling pathways, TFs, glycolytic genes, and glycolysis-related pathways.

Using TFBSTools and JASPAR database, we predicted E2F1, E2F4, and E2F6 to be possible TFs promoting the expression of *PFKP*, *ENO3*, and *GAPDH*. Moreover, hematopoietic cell-specific ChIP-seq data further supported E2F6 as a TF regulating the expression of *PFKP*, *ENO3*, and *GAPDH* (**Figure S9**), which was in accordance with the positive correlation between E2F6 and their expressions (**Figure S7B**) **(** 18). Furthermore, we confirmed that T cells with higher E2F6 expression harbored increased expression of *PFKP*, *ENO3*, and *GAPDH* in our patient cohort (**Figure S10**). Taken together, these results suggest that TCR, mTORC1, and MYC signaling pathways might promote the expression of *PFKP*, *ENO3*, and *GAPDH* through TF E2F6 to activate glycolysis and its related pathways in alloreactive T cells during aGvHD development (**Figure 6**).

## Discussion

The benefits of allo-HSCT are challenged by GvHD, which is one of the most common causes of treatment-related mortality in the early phase after allo-HSCT. Although glucocorticoid is demonstrated as the frontline therapy for aGvHD, 40%–50% is steroid refractory and resulted in 60%–80% mortality. The optimal treatment for steroid-refractory aGvHD is still under exploration. Thus, identification and characterization of novel targets of aGvHD are important for therapy revolution. There is growing evidence that metabolomics play a role in different aspects of aGvHD (30). Therefore, to elucidate the metabolic pathways employed by T cells is important to deepening our understanding of aGvHD pathophysiology. However, the comprehensive metabolic network of allogeneic T cells in aGvHD setting is largely underestimated, particularly in humans.

Since the transcriptional expression patterns of metabolic pathways have been demonstrated to reflect metabolic activities definitely (14), we integrated publicly accessible GEO datasets of human alloreactive T cells and systematically characterized the metabolic programming in correlation with aGvHD based on the expressional heterogeneity of metabolic genes. To the best of our knowledge, this global perspective has not been used to study aGvHD previously. We found that carbohydrate metabolism was the most significantly enriched metabolic super-pathway in alloreactive T cells in the pre-aGvHD state, and glycolysis had the most extensive correlation with other metabolic sub-pathways (**Figure 1**). High predictive value of aGvHD risk model constructed by well-known glycolytic genes further iterated the crucial role of glycolysis in alloreactive T cells (**Figure 5**). In compliance with other *in vitro* and *in vivo* studies, activated glycolytic activity is increased in T cells to meet their biomass demand for synthesis of macromolecules during robust proliferation (9, 31).

The modality of metabolic reprogramming in alloreactive T cells is controversial across different GvHD models. Gatza et al. (32) found that alloreactive T cells, in response to allo-antigens, greatly increased both glycolysis and oxidative phosphorylation, and the activation of oxidative phosphorylation was due to an increase of fatty acid oxidation *via* TCA cycle compared to naive T cells (rather than donor T cells in the syngeneic recipients) in unirradiated murine GvHD model. Compared to general T-cell activation, alloreactive T cell experiences the inflammatory milieu of pretransplant conditioning and reconstitution of the immune system. As to distinguish alloreactive from homeostatically proliferating T cells, syngeneic T cells are better negative control than naive T cells (33). Byersdorfer et al. (10) showed that alloreactive T cells used fatty acid oxidation as the major fuel source during activation. However, Nguyen et al. (9) demonstrated that inhibition of fatty acid oxidation by etomoxir was not enough to significantly affect alloreactive T-cell proliferation. Given the great differences among the models, the abovementioned studies might not be able to describe metabolic reprogramming of human alloreactive T cells well in a pre-aGvHD state. Our previous study integrated the metabolic and transcriptomic analyses and discovered increased glycerophospholipid metabolism in a pre-aGvHD state (8). However, the study used a non-targeted approach (liquid chromatography-mass spectrometry) to detect plasma metabolites and the transcriptomic data were derived from mononuclear cells in peripheral blood. In the present study, we found that CD4 memory T cell was the top subset that positively correlated with glycolysis activation (**Figures 3A, B** **)**. Alloreactive CD4 effector memory T cell is the predominant pathogenic subset and highly glycolytic in our clinical observation (Zhang et al., unpublished data) and allo-HSCT murine model (34). Activated T-cell proliferation and migration signatures were positively associated with glycolytic activation (**Figures S3A, B**). These results indicated the pivotal function of glycolytic metabolism in specified T-cell subset activation and immune function in pre-aGvHD state.

Identification of crucial signaling pathways and TFs that promote glycolysis could help us to discover potential targets to suspend the uncontrolled activation of alloreactive T cells (9, 35). Metabolic reprogramming occurs generally downstream of TCR and the co-stimulatory receptor (such as CD28) signaling following interaction between T cells and APCs in autoimmune disease, which activates mTORC1 and MYC signaling. Consequently, T-cell metabolism shifts from fatty acid and pyruvate oxidation in steady state toward glycolysis during activation (27). mTORC1, a key driver of cell metabolism (36–39), integrated microenvironment cues with T-cell metabolism and activation state. E2F TF family, which is regulated by both MYC and mTOR signaling (40, 41) and promotes glycolytic metabolism (42) as reported, was also activated. The activation of E2F family might bridge MYC and mTORC1 signaling with glycolytic activation (**Figure 6**). In murine models, alloreactive T cells showed higher expression of glucose transporters Glut1 and Glut3 and glycolytic enzymes hexokinase and lactate dehydrogenase. Furthermore, AMPK and mTOR pathways were activated and subsequently resulted in greater increase of glucose uptake and glycolysis than syngeneic T cells (9, 32). The context-specific programming observed in alloreactive T cells is unique and different from other physiological processes.

There are limitations in our study. First, although we integrated and normalized the transcriptomic data, bias and variabilities might also exist due to technical limitations, such as dye effect, hybridization artifacts, and between-sample differences (i.e., different sample acquisition times). Second, due to the limited information provided by the original studies, we could not correlate metabolism reprogramming with patient background, aGvHD severity, and treatment outcome. Moreover, we could not discriminate T-cell subsets, such as Teff, Treg, and Tm, for detailed metabolic signatures. However, with our integrative and robust bioinformatic analyses, we identified glycolysis contributing to the activated allogeneic CD4 memory T-cell subset and T-cell proliferation and migration in a pre-aGvHD state, as well as TCR, mTORC1, and MYC signaling pathways and E2F6 might be “master regulators” of glycolytic activation. Moreover, the increased CD4 memory T-cell subset and glycolytic activation in pre-aGvHD state were validated in our patient cohort. These results are objective and meaningful to provide clues for aGvHD prophylaxis and treatment.

Taken together, we identified metabolic pathways that were enriched in alloreactive T cells in a pre-aGvHD state and highlighted T-cell subsets, T-cell immune functions, key signaling pathways, and TFs that were closely linked with glycolysis activation. The hypothesis of alloreactive T-cell metabolic reprogramming during aGvHD pathogenesis (especially a pre-aGvHD state) is presented in **Figure 6**. Our data emphasize the pivotal function of glycolysis in alloreactive T-cell activation in a pre-aGvHD state, and glycolysis would be the potential target for GvHD treatment.

## Data Availability Statement

Publicly available datasets were analyzed in this study. This data can be found here: [https://www.ncbi.nlm.nih.gov/geo/query/acc.cgi?acc=GSE4624. https://www.ncbi.nlm.nih.gov/geo/query/acc.cgi?acc=GSE73809]. The raw data supporting the conclusions of this article will be made available by the authors, without undue reservation.

## Ethics Statement

The studies involving human participants were reviewed and approved by Ruijin Hospital Ethics Committee, Shanghai Jiao Tong Univerity School of Medicine. The patients/participants provided their written informed consent to participate in this study.

## Author Contributions

ZKP, AJH, and YH analyzed the data and wrote the article. ZLZ, CHJ, LXW, KQ, and SJZ analyzed the data. JMW and XXH designed the study, analyzed the data, and wrote the article. All authors have read and approved the final version of the article.

## Funding

This work was supported by the National Natural Science Foundation of China (NSFC 82170206, 81770160, 81870143).

## Conflict of Interest

The authors declare that the research was conducted in the absence of any commercial or financial relationships that could be construed as a potential conflict of interest.

## Publisher’s Note

All claims expressed in this article are solely those of the authors and do not necessarily represent those of their affiliated organizations, or those of the publisher, the editors and the reviewers. Any product that may be evaluated in this article, or claim that may be made by its manufacturer, is not guaranteed or endorsed by the publisher.

## References

[1] Ferrara JL Levine JE Reddy P Holler E. Graft-Versus-Host Disease. Lancet (2009) 373(9674):1550–61. doi: 10.1016/S0140-6736(09)60237-3 PMC2735047 19282026

[2] Phelan R Arora M Chen M. Current Use and Outcome of Hematopoietic Stem Cell Transplantation: CIBMTR US Summary Slides. (2020). Available at: https://www.cibmtr.org/ReferenceCenter/SlidesReports/SummarySlides/Pages/index.aspx.

[3] Srinagesh HK Levine JE Ferrara JLM. Biomarkers in Acute Graft-Versus-Host Disease: New Insights. Ther Adv Hematol (2019) 10:2040620719891358. doi: 10.1177/2040620719891358 31839920 PMC6893923

[4] Braun LM Zeiser R. Kinase Inhibition as Treatment for Acute and Chronic Graft-Versus-Host Disease. Front Immunol (2021) 12:760199. doi: 10.3389/fimmu.2021.760199 34868001 PMC8635802

[5] Zewde MG Morales G Gandhi I Ozbek U Aguayo-Hiraldo P Ayuk F. Evaluation of Elafin as a Prognostic Biomarker in Acute Graft-Versus-Host Disease. Transplant Cell Ther (2021) 27(12):988.e1-7. doi: 10.1016/j.jtct.2021.08.021 PMC8671218 34474163

[6] Aziz MD Shah J Kapoor U Dimopoulos C Anand S Augustine A. Disease Risk and GVHD Biomarkers can Stratify Patients for Risk of Relapse and Nonrelapse Mortality Post Hematopoietic Cell Transplant. Leukemia (2020) 34(7):1898–906. doi: 10.1038/s41375-020-0726-z PMC7332389 32020045

[7] Srinagesh HK Ozbek U Kapoor U Ayuk F Aziz M Ben-David K. The MAGIC Algorithm Probability is a Validated Response Biomarker of Treatment of Acute Graft-Versus-Host Disease. Blood Adv (2019) 3(23):4034–42. doi: 10.1182/bloodadvances.2019000791 PMC6963240 31816061

[8] Liu Y Huang A Chen Q Chen X Fei Y Zhao X. A Distinct Glycerophospholipid Metabolism Signature of Acute Graft Versus Host Disease With Predictive Value. JCI Insight (2019) 4(16):e129494. doi: 10.1172/jci.insight.129494 PMC6777804 31343987

[9] Nguyen HD Chatterjee S Haarberg KM Wu Y Bastian D Heinrichs J. Metabolic Reprogramming of Alloantigen-Activated T Cells After Hematopoietic Cell Transplantation. J Clin Invest (2016) 126(4):1337–52. doi: 10.1172/JCI82587 PMC4811142 26950421

[10] Byersdorfer CA Tkachev V Opipari AW Goodell S Swanson J Sandquist S. Effector T Cells Require Fatty Acid Metabolism During Murine Graft-Versus-Host Disease. Blood (2013) 122(18):3230–7. doi: 10.1182/blood-2013-04-495515 PMC3814737 24046012

[11] Leek JT Johnson WE Parker HS Jaffe AE Storey JD. The Sva Package for Removing Batch Effects and Other Unwanted Variation in High-Throughput Experiments. Bioinformatics (2012) 28(6):882–3. doi: 10.1093/bioinformatics/bts034 PMC3307112 22257669

[12] Ritchie ME Phipson B Wu D Hu Y Law CW Shi W. Limma Powers Differential Expression Analyses for RNA-Sequencing and Microarray Studies. Nucleic Acids Res (2015) 43(7):e47. doi: 10.1093/nar/gkv007 25605792 PMC4402510

[13] Hanzelmann S Castelo R Guinney J. GSVA: Gene Set Variation Analysis for Microarray and RNA-Seq Data. BMC Bioinf (2013) 14:7. doi: 10.1186/1471-2105-14-7 PMC3618321 23323831

[14] Peng X Chen Z Farshidfar F Xu X Lorenzi PL Wang Y. Molecular Characterization and Clinical Relevance of Metabolic Expression Subtypes in Human Cancers. Cell Rep (2018) 23(1):255–69.e4. doi: 10.1016/j.celrep.2018.03.077 29617665 PMC5916795

[15] Fabregat A Sidiropoulos K Garapati P Gillespie M Hausmann K Haw R. The Reactome Pathway Knowledgebase. Nucleic Acids Res (2016) 44(D1):D481–7. doi: 10.1093/nar/gkv1351 PMC4702931 26656494

[16] Newman AM Steen CB Liu CL Gentles AJ Chaudhuri AA Scherer F. Determining Cell Type Abundance and Expression From Bulk Tissues With Digital Cytometry. Nat Biotechnol (2019) 37(7):773–82. doi: 10.1038/s41587-019-0114-2 PMC6610714 31061481

[17] Tan G Lenhard B. TFBSTools: An R/bioconductor Package for Transcription Factor Binding Site Analysis. Bioinformatics (2016) 32(10):1555–6. doi: 10.1093/bioinformatics/btw024 PMC4866524 26794315

[18] Zheng R Wan C Mei S Qin Q Wu Q Sun H. Cistrome Data Browser: Expanded Datasets and New Tools for Gene Regulatory Analysis. Nucleic Acids Res (2019) 47(D1):D729–D35. doi: 10.1093/nar/gky1094 PMC6324081 30462313

[19] Baron C Somogyi R Greller LD Rineau V Wilkinson P Cho CR. Prediction of Graft-Versus-Host Disease in Humans by Donor Gene-Expression Profiling. PloS Med (2007) 4(1):e23. doi: 10.1371/journal.pmed.0040023 17378698 PMC1796639

[20] Furlan SN Watkins B Tkachev V Flynn R Cooley S Ramakrishnan S. Transcriptome Analysis of GVHD Reveals Aurora Kinase A as a Targetable Pathway for Disease Prevention. Sci Transl Med (2015) 7(315):315ra191. doi: 10.1126/scitranslmed.aad3231 PMC4876606 26606970

[21] Kim NH Cha YH Lee J Lee SH Yang JH Yun JS. Snail Reprograms Glucose Metabolism by Repressing Phosphofructokinase PFKP Allowing Cancer Cell Survival Under Metabolic Stress. Nat Commun (2017) 8:14374. doi: 10.1038/ncomms14374 28176759 PMC5309788

[22] Park C Lee Y Je S Chang S Kim N Jeong E. Overexpression and Selective Anticancer Efficacy of ENO3 in STK11 Mutant Lung Cancers. Mol Cells (2019) 42(11):804–9. doi: 10.14348/molcells.2019.0099 PMC6883975 31697874

[23] Kornberg MD Bhargava P Kim PM Putluri V Snowman AM Putluri N. Dimethyl Fumarate Targets GAPDH and Aerobic Glycolysis to Modulate Immunity. Science (2018) 360(6387):449–53. doi: 10.1126/science.aan4665 PMC5924419 29599194

[24] Liao ST Han C Xu DQ Fu XW Wang JS Kong LY. 4-Octyl Itaconate Inhibits Aerobic Glycolysis by Targeting GAPDH to Exert Anti-Inflammatory Effects. Nat Commun (2019) 10(1):5091. doi: 10.1038/s41467-019-13078-5 31704924 PMC6841710

[25] Zhong XY Yuan XM Xu YY Yin M Yan WW Zou SW. CARM1 Methylates GAPDH to Regulate Glucose Metabolism and Is Suppressed in Liver Cancer. Cell Rep (2018) 24(12):3207–23. doi: 10.1016/j.celrep.2018.08.066 30232003

[26] Bantug GR Galluzzi L Kroemer G Hess C. The Spectrum of T Cell Metabolism in Health and Disease. Nat Rev Immunol (2018) 18(1):19–34. doi: 10.1038/nri.2017.99 28944771

[27] Sharabi A Tsokos GC. T Cell Metabolism: New Insights in Systemic Lupus Erythematosus Pathogenesis and Therapy. Nat Rev Rheumatol (2020) 16(2):100–12. doi: 10.1038/s41584-019-0356-x 31949287

[28] Menk AV Scharping NE Moreci RS Zeng X Guy C Salvatore S. Early TCR Signaling Induces Rapid Aerobic Glycolysis Enabling Distinct Acute T Cell Effector Functions. Cell Rep (2018) 22(6):1509–21. doi: 10.1016/j.celrep.2018.01.040 PMC5973810 29425506

[29] Saxton RA Sabatini DM. mTOR Signaling in Growth, Metabolism, and Disease. Cell (2017) 169(2):361–71. doi: 10.1016/j.cell.2017.03.035 28388417

[30] Kumari R Palaniyandi S Hildebrandt GC. Metabolic Reprogramming-A New Era How to Prevent and Treat Graft Versus Host Disease After Allogeneic Hematopoietic Stem Cell Transplantation Has Begun. Front Pharmacol (2020) 11:588449. doi: 10.3389/fphar.2020.588449 33343357 PMC7748087

[31] Wang R Dillon CP Shi LZ Milasta S Carter R Finkelstein D. The Transcription Factor Myc Controls Metabolic Reprogramming Upon T Lymphocyte Activation. Immunity (2011) 35(6):871–82. doi: 10.1016/j.immuni.2011.09.021 PMC3248798 22195744

[32] Gatza E Wahl DR Opipari AW Sundberg TB Reddy P Liu C. Manipulating the Bioenergetics of Alloreactive T Cells Causes Their Selective Apoptosis and Arrests Graft-Versus-Host Disease. Sci Transl Med (2011) 3(67):67ra8. doi: 10.1126/scitranslmed.3001975 PMC3364290 21270339

[33] Brown RA Byersdorfer CA. Metabolic Pathways in Alloreactive T Cells. Front Immunol (2020) 11:1517. doi: 10.3389/fimmu.2020.01517 32793207 PMC7393946

[34] Assmann JC Farthing DE Saito K Maglakelidze N Oliver B Warrick KA. Glycolytic Metabolism of Pathogenic T Cells Enables Early Detection of GVHD by 13C-MRI. Blood (2021) 137(1):126–37. doi: 10.1182/blood.2020005770 PMC7808015 32785680

[35] Nguyen HD Kuril S Bastian D. Yu XZ. T-Cell Metabolism in Hematopoietic Cell Transplantation. Front Immunol (2018) 9:176. doi: 10.3389/fimmu.2018.00176 29479351 PMC5811499

[36] Kim J Guan KL. mTOR as a Central Hub of Nutrient Signalling and Cell Growth. Nat Cell Biol (2019) 21(1):63–71. doi: 10.1038/s41556-018-0205-1 30602761

[37] Liu GY Sabatini DM. mTOR at the Nexus of Nutrition, Growth, Ageing and Disease. Nat Rev Mol Cell Biol (2020) 21(4):183–203. doi: 10.1038/s41580-019-0199-y 31937935 PMC7102936

[38] Huang H Long L Zhou P Chapman NM Chi H. mTOR Signaling at the Crossroads of Environmental Signals and T-Cell Fate Decisions. Immunol Rev (2020) 295(1):15–38. doi: 10.1111/imr.12845 32212344 PMC8101438

[39] Long L Wei J Lim SA Raynor JL Shi H Connelly JP. CRISPR Screens Unveil Signal Hubs for Nutrient Licensing of T Cell Immunity. Nature (2021) 600(7888):308–13. doi: 10.1038/s41586-021-04109-7 PMC8887674 34795452

[40] Michaloglou C Crafter C Siersbaek R Delpuech O Curwen JO Carnevalli LS. Combined Inhibition of mTOR and CDK4/6 Is Required for Optimal Blockade of E2F Function and Long-Term Growth Inhibition in Estrogen Receptor-Positive Breast Cancer. Mol Cancer Ther (2018) 17(5):908–20. doi: 10.1158/1535-7163.MCT-17-0537 PMC6485624 29483206

[41] Bretones G Delgado MD Leon J. Myc and Cell Cycle Control. Biochim Biophys Acta (2015) 1849(5):506–16. doi: 10.1016/j.bbagrm.2014.03.013 24704206

[42] Murata K Fang C Terao C Giannopoulou EG Lee YJ Lee MJ. Hypoxia-Sensitive COMMD1 Integrates Signaling and Cellular Metabolism in Human Macrophages and Suppresses Osteoclastogenesis. Immunity (2017) 47(1):66–79.e5. doi: 10.1016/j.immuni.2017.06.018 28723554 PMC5568808

