# Supplementary material for: Metabolic Reprogramming of Alloreactive T Cells Through TCR/MYC/mTORC1/E2F6 Signaling in aGvHD Patients
Source: Front Immunol. 2022 Mar 25;13:850177. doi: 10.3389/fimmu.2022.850177 (PMC8989838; doi:10.3389/fimmu.2022.850177)
Supplement: Supplementary file 1 [file DataSheet_1.pdf]

Table S1. Details of aGvHD associated studies and transcriptomic datasets from GEO database

Series accession	Platform	Sample size (n)	Sampling time	Upregulated DEGs	Downregulated DEGs	Sex	Age (y)
GSE4624	GPL3639	Total: 61<br>pre-aGvHD: 37<br>non-aGvHD: 24	+0	760	351	NA	NA
GSE73809	GPL17586	Total: 24<br>pre-aGvHD: 11<br>non-aGvHD: 13	+28~84	1728	876	NA	22-73

aGvHD: acute graft-versus-host disease; non-aGvHD: no acute graft-versus-host disease; DEGs: differentially expressed genes; NA: not available.

Table S2. List of genes invloved in the seven metabolic super signatures surveyed.

Amino acid	Carbohydrate	Energy	Lipid	Nucleotide	TCA cycle	Vitamin&cofactor
SLC3A2	*PPP2R1A*	*PPP2R1A*	*HSD3B1*	*NT5C2*	*ACO2*	*NT5E*
GOT1	*PPP2R1B*	*PPP2R1B*	*HSD3B2*	*NT5E*	*NDUFAB1*	*ABCD4*
GOT2	*PGD*	*ADRA2A*	*AP2A1*	*ADA*	*ATP5G1*	*AOX1*
ACAD8	*PGLS*	*PRKAA2*	*AP2A2*	*ADK*	*ATP5G2*	*AKR1B10*
ACADSB	*AAAS*	*PRKAB2*	*AP2B1*	*AGXT2*	*ATP5G3*	*AKR1C1*
NDUFAB1	*GOT1*	*PRKAG2*	*AP2M1*	*AMPD1*	*ATP5A1*	*AKR1C3*
ASPA	*GOT2*	*ACLY*	*A2M*	*AMPD2*	*ATP5B*	*AKR1C4*
AGXT2	*AKR1A1*	*CHRM3*	*AP2S1*	*AMPD3*	*ATP5D*	*APOA1*
GPT	*AKR1B1*	*SLC25A4*	*PRKAA2*	*APRT*	*ATP5E*	*APOA2*
ARG2	*ALDOA*	*SLC25A5*	*PRKAB2*	*UPB1*	*ATP5F1*	*APOA4*
ARG1	*ALDOB*	*SLC25A6*	*PRKAG2*	*CAT*	*ATP5C1*	*APOB*
ASL	*ALDOC*	*ADIPOQ*	*ABCB11*	*CDA*	*ATP5I*	*APOC2*
ASNS	*AMY2B*	*ARL2*	*ABCA1*	*DCK*	*ATP5J2*	*APOC3*
ASS1	*AMY2A*	*CACNA1A*	*ABCG5*	*DCTD*	*ATP5L*	*APOE*
SAT1	*AMY1A*	*CACNA1E*	*ABCG8*	*DGUOK*	*ATP5O*	*APOM*
TAT	*NAGLU*	*CACNB2*	*DBI*	*DPYD*	*ATP5H*	*HLCS*
BCAT2	*ARSB*	*CACNB3*	*ACADL*	*DPYS*	*ATP5J*	*BST1*
BCAT1	*B3GAT1*	*ACACB*	*ACADM*	*ENTPD1*	*ATP5S*	*BTD*
BCKDK	*B3GAT2*	*ADCY1*	*ACADS*	*ENTPD2*	*BSG*	*MTHFD1*
BHMT	*B3GAT3*	*ADCY2*	*ACADVL*	*ENTPD3*	*SDHC*	*CD38*
BBOX1	*B3GNT1*	*ADCY3*	*ACHE*	*ENTPD4*	*NDUFAF1*	*CTRC*
CBS	*B3GNT2*	*ADCY4*	*ACLY*	*ENTPD5*	*CS*	*ACACB*
CNDP2	*B3GNT3*	*ADCY5*	*SCD*	*ENTPD6*	*COX7A2L*	*CLPS*
CTH	*B4GALT1*	*ADCY6*	*NDUFAB1*	*GLRX*	*COX5A*	*CYP24A1*
CPS1	*B4GALT2*	*ADCY7*	*AGPS*	*GMPR*	*COX5B*	*CYP27B1*
CRYM	*B4GALT3*	*ADCY8*	*PLIN2*	*GMPR2*	*COX6A1*	*CYP8B1*
CSAD	*B4GALT4*	*ADCY9*	*FDXR*	*GPX1*	*COX6B1*	*CTRB1*
CDO1	*B4GALT5*	*PFKFB1*	*FDX1*	*GMPS*	*COX6C*	*CYB5A*
HIBADH	*B4GALT6*	*FASN*	*GLA*	*GDA*	*COX7B*	*SLC25A19*
ALDH7A1	*B4GALT7*	*GNA11*	*AHR*	*HPRT1*	*COX7C*	*DHFR*
AMD1	*GLB1*	*GNA14*	*AKR1C1*	*IMPDH1*	*COX8A*	*FASN*
HDC	*GUSB*	*GNA15*	*AKR1C2*	*IMPDH2*	*COX11*	*SLC19A1*
ODC1	*CALM1*	*GNAS*	*AKR1C3*	*AK1*	*COX4I1*	*FOLR2*
DDC	*CD44*	*GNB1*	*AKR1C4*	*AK2*	*CYC1*	*ALDH1L1*
ALDH9A1	*SLC25A12*	*GNB2*	*AKR1D1*	*AK5*	*CYCS*	*MTHFS*
GLUD1	*SLC25A13*	*GNB3*	*ALB*	*CMPK1*	*SDHA*	*SLC25A16*
GLUD2	*CRYL1*	*GNB4*	*AKR1B1*	*GUK1*	*SDHB*	*GPHN*
QDPR	*DERA*	*GNGT1*	*ABCD1*	*TK2*	*SDHD*	*SHMT1*
SLC25A10	*ALDH1A1*	*GNG3*	*AMACR*	*DTYMK*	*DLD*	*SHMT2*
DLD	*SORD*	*GNG4*	*AGT*	*NME1*	*ETFA*	*GPC1*
DBH	*SLC25A10*	*GNG5*	*ANGPTL3*	*NME2*	*ETFB*	*GPC3*
SHFM1	*SLC26A2*	*GNG7*	*ANGPTL4*	*NME4*	*FH*	*GPC4*
FAH	*ENO1*	*GNG8*	*APOA1*	*NUDT5*	*IDH3A*	*GPC5*
IL4I1	*ENO3*	*GNG10*	*APOA2*	*PNP*	*IDH3B*	*GPC6*
FOLH1	*ENO2*	*GNG11*	*APOA4*	*PPAT*	*IDH3G*	*GSTO1*
FTCD	*EXT1*	*GNG12*	*APOB*	*GART*	*IDH2*	*SLC2A1*
GAMT	*EXT2*	*GNG13*	*APOC1*	*PFAS*	*LDHA*	*SLC2A3*
GATM	*FBP1*	*GNGT2*	*APOC2*	*PAICS*	*LDHB*	*GIF*
GCDH	*FBP2*	*GNAI1*	*APOC3*	*ADSL*	*LDHC*	*LDLR*
GCSH	*PFKFB1*	*GNAI2*	*APOC4*	*ATIC*	*LDHAL6B*	*LGMN*
GLDC	*PFKFB2*	*GNAQ*	*APOE*	*ADSS*	*GLO1*	*LPL*
AMT	*PFKFB3*	*GCGR*	*APOF*	*CAD*	*LRPPRC*	*PNLIP*
CGA	*PFKFB4*	*GCG*	*ACER3*	*UMPS*	*MDH2*	*LRP1*
GNMT	*FGF21*	*FFAR1*	*LPA*	*DHODH*	*SLC16A1*	*LRP2*
GLUL	*FMOD*	*SLC2A1*	*ARF1*	*RRM1*	*SLC16A8*	*MCCC1*

GLS	GAPDH	SLC2A2	ARF3	RRM2	SLC16A3	MCCC2
GLS2	GAPDHS	INS	ARNT2	TXN	NDUFA7	MTR
SHMT1	G6PD	ITPR3	ARNT	TXNRD1	NDUFC2	SLC25A32
GCLM	GPI	KCNJ11	ARSA	TYMP	NDUFA12	MOCS3
GCLC	G6PC	PRKAR1A	ARSB	TYMS	NDUFB3	ABCC1
HAO1	GALNS	PRKAR1B	ARSD	UCK1	NDUFA6	MOCS2
HSD17B10	GALK1	PRKAR2A	ARSE	UCK2	NDUFB4	MTHFD2
HGD	GALT	PRKAR2B	ARSF	UPP1	NDUFA13	MTHFR
HNMT	GALE	PRKACA	ASAH1	XDH	NDUFB6	MTRR
HPD	GCKR	PRKACB	SMPD1	TK1	NDUFB7	MUT
HAL	AGL	PRKACG	ACOT7	ITPA	NDUFB9	QPRT
UROC1	GNS	PRKCA	CEL	AK7	NDUFA2	COASY
IDO1	GLCE	PKLR	BDH1	NT5C	NDUFA3	CYB5R3
INMT	GBE1	MARCKS	GLB1	NT5M	NDUFB8	NNMT
DIO3	GYG1	MLX	BHMT	ADSSL1	NDUFB10	ENPP1
IVD	GNPDA1	PPP2CA	BMX	UPP2	NDUFB2	ENPP2
GCAT	GPC1	PPP2CB	CYP11A1	NT5C1A	NDUFC1	ENPP3
CKB	GPC3	PLCB1	CYP39A1	NT5C1B	NDUFA1	NAMPT
CKM	GPC4	PLCB2	ACOT12	DUT	NDUFB1	PCCA
CKMT2	GPC5	PLCB3	CRAT	NUDT13	NDUFS5	PCCB
CKMT1A	GPC6	AGPAT1	B4GALNT1	POMP	NDUFB5	PDXK
KYNU	SLC2A1	RAP1A	ACOX1	NUDT1	NNT	HSPG2
SLC7A5	SLC2A2	AHCYL1	ACOX2	ADAL	NDUFS1	PTGS2
LIAS	SLC2A3	STK11	ACOX3	DDX31	NDUFV1	PANK2
LIPT1	SLC2A4	SYT5	CAV1	ENTPD7	NDUFS2	PANK3
GSTZ1	SLC2A5	TALDO1	NFYB	ENTPD8	NDUFA10	PANK4
SLC45A2	GYG2	TKT	CREBBP	GSR	NDUFA9	ACP5
AIMP1	GYS1	VAMP2	CD36	LHPP	NDUFA5	NADK
AIMP2	GYS2	MLXIPL	CDS1	NUDT15	NDUFS3	PARP4
EEF1E1	HAS1	ADIPOR1	CDS2	NUDT16	NDUFV2	PTGIS
MCCC1	HAS2	ADIPOR2	UGCG	CTPS2	NDUFS8	PC
MCCC2	HAS3	GNG2	CETP	RRM2B	NDUFS7	RBP1
MTR	HEXA	ADRA2C	ACOT9	DCTPP1	NDUFA4	RBP2
MAT1A	HEXB	CACNA2D2	CCNC	NUDT18	NDUFS6	RBP4
ALDH6A1	HMMR	ARL2BP	UGT8	NUDT9	NDUFV3	SDC1
MTAP	NDST1	CACNA1C	BCHE		NDUFA8	SDC2
MTRR	NDST2	GLP1R	CIDEA		NDUFS4	SDC3
NAALAD2	HK1	AKAP5	CLOCK		OGDH	SDC4
NNMT	HK2	KCNS3	CPNE1		DLST	SLC5A6
NQO1	HK3	IQGAP1	CPNE3		DLAT	LRP12
OAT	HYAL2	KCNB1	CPNE6		PDHA1	SLC23A1
OAZ2	IDS	KCNG2	CPNE7		PDHB	SLC23A2
OAZ3	IDUA	GNB5	ACACB		PDHX	TCN1
OAZ1	NUP160	RAPGEF3	POMC		PDK1	TCN2
DBT	PFKM	SNAP25	CLPS		PDK2	SLC19A2
BCKDHA	PFKL	STXBP1	COQ3		PDK3	PRSS1
BCKDHB	PFKP	CACNA1D	COQ6		PDK4	PRSS3
AZIN1	PRKACA	ABCC8	COQ7		PDP1	TTR
SLC25A21	PRKACB	ACACA	CYP1A1		PDP2	VNN1
OGDH	PRKACG	ITPR2	CYP1A2		PPARD	VNN2
DLST	KERA	ITPR1	CYP1B1		RXRA	GC
DLAT	KHK	PPP2R5D	CYP27A1		SUCLA2	LRAT
PDHA1	PHKA1	STX1A	CYP46A1		SUCLG2	AMN
PDHB	PHKA2	RAPGEF4	CYP4B1		SCO1	PARP9
PDHX	PHKB	KCNC2	CYP4A11		SCO2	BCMO1
SLC25A15	PHKG1		CYP51A1		SUCLG1	BCO2
SLC25A2	PHKG2		CYP7A1		SURF1	GSTO2
OTC	PRPS1		CYP7B1		TRAP1	MMAA

DAO	*PRPS2*	*CYP8B1*	*UCP1*	*MMAB*
DDO	*PRPS1L1*	*CYP2C8*	*UCP2*	*NMNAT1*
PAH	*PKLR*	*CYP2C9*	*SLC25A27*	*NMNAT2*
PCBD1	*LALBA*	*CYP2C19*	*UQCRC1*	*PNPO*
PNMT	*LCT*	*CYP4F2*	*UQCRC2*	*RDH11*
PAPSS1	*LUM*	*CYP4F3*	*UQCRB*	*RFK*
PYCR1	*GAA*	*CYP4F11*	*UQCRH*	*THTPA*
PRODH	*MAN2B1*	*CYP2J2*	*UQCRFS1*	*AGRN*
PSMC5	*MAN2B2*	*CYP11B1*	*UQCRQ*	*LRP10*
PSMA8	*MAN2C1*	*CYP11B2*	*UQCR10*	*LRP8*
ALDH4A1	*SLC25A11*	*CYP21A2*	*UQCR11*	*NMNAT3*
OCA2	*MANBA*	*CPT1A*	*ACAD9*	*PPCS*
RPS4Y2	*MDH1*	*CPT2*	*NDUFAF4*	*TPK1*
SLC6A7	*MDH2*	*CYP17A1*	*NUBPL*	*VKORC1*
SLC6A8	*MGAM*	*CPT1B*	*NDUFB11*	*PPCDC*
SLC6A11	*OGN*	*CYP19A1*	*COX18*	*FLAD1*
SLC6A12	*ABCC5*	*MED17*	*TACO1*	*NADSYN1*
AHCY	*NUP107*	*MED7*	*LDHAL6A*	*FPGS*
SECISBP2	*NUP153*	*SPTLC3*	*NDUFA11*	*AASDHPPT*
EEFSEC	*NUP214*	*FITM2*	*ECSIT*	*SLC19A3*
PHGDH	*SLC9A1*	*CTGF*	*NDUFAF3*	*SLC46A1*
PSPH	*NUP37*	*PCYT1A*	*COX16*	*CYP2R1*
PSAT1	*NUP43*	*PCYT1B*	*COX19*	*APOA1BP*
SLC5A5	*NUP50*	*MID1IP1*	*COQ10A*	*ALDH1L2*
AANAT	*NUP62*	*ALDH7A1*	*COQ10B*	*MMADHC*
PIPOX	*NUP88*	*DAB1*	*D2HGDH*	*CD320*
AGMAT	*OMD*	*GNPAT*	*ADHFE1*	*CTRB2*
SRM	*PPP2CA*	*MLYCD*	*L2HGDH*	*CUBN*
SMS	*PPP2CB*	*DECR1*	*NDUFAF2*	*DHFRL1*
AGXT	*HSPG2*	*DGAT1*	*TMEM126B*	*GPC2*
SQRDL	*ACAN*	*ALDH3B1*	*PDPR*	*GPIHBP1*
SUOX	*NCAN*	*ALDH3B2*	*SLC25A14*	*LMBRD1*
DARS	*VCAN*	*HSD17B1*	*UCP3*	*MMACHC*
EPRS	*PGK1*	*HSD17B2*	*ETFDH*	*MOCOS*
IARS	*PGK2*	*HSD17B3*	*HAGH*	*NFS1*
KARS	*PGM1*	*HSD17B4*		*NUDT12*
MARS	*BGN*	*HSD17B8*		*PARP10*
QARS	*DCN*	*DHCR7*		*PARP14*
RARS	*PYGL*	*CBR1*		*PARP16*
SARS	*PYGM*	*DHCR24*		*PARP6*
TDO2	*PYGB*	*HSD11B1*		*PARP8*
SERINC3	*PGAM1*	*HSD11B2*		*PDZD11*
SERINC1	*PGAM2*	*EBP*		*RFT1*
ACAT1	*BPGM*	*HADHA*		*PLB1*
TST	*PCK1*	*HADHB*		*NAPRT1*
TMLHE	*PCK2*	*ECHS1*		*SLC22A13*
TXNRD1	*PAPSS1*	*EHHADH*		*UBIAD1*
TSHB	*PRELP*	*ETNK1*		*VKORC1L1*
TYRP1	*PC*	*ETNK2*		*MTHFD2L*
DCT	*RAE1*	*ELOVL1*		*SLC5A8*
TYR	*RANBP2*	*ELOVL2*		*CARKD*
PSMD14	*RPIA*	*ELOVL3*		*MTHFD1L*
NAGS	*SLC26A1*	*ELOVL4*		*ACACA*
HIBCH	*SDC1*	*MVD*		*PANK1*
AASS	*SDC2*	*TM7SF2*		*MOCS1*
AADAT	*SDC3*	*SQLE*		
KMO	*SDC4*	*LSS*		
ACMSD	*ST3GAL1*	*ESRRA*		

RPS10	*ST3GAL2*	*FAAH*
RPS11	*ST3GAL4*	*FABP4*
RPS12	*ST3GAL3*	*FABP7*
RPS13	*SLC5A1*	*FABP5*
RPS14	*SLC5A2*	*FABP3*
RPS15	*SLC5A3*	*FABP2*
RPS16	*SLC5A4*	*FABP1*
RPS17	*SGSH*	*FASN*
RPS18	*SI*	*FDFT1*
RPS19	*TALDO1*	*FGF21*
RPS15A	*TKT*	*ALOX5AP*
RPS2	*TPR*	*FDPS*
RPS20	*TREH*	*CIDEC*
RPS21	*SLC25A1*	*FURIN*
RPS23	*NUP155*	*KDSR*
RPS24	*RPS27A*	*FYN*
RPS25	*UBA52*	*PIKFYVE*
RPS26	*CHIA*	*G0S2*
RPS27	*CHIT1*	*GALC*
RPS27A	*NUP133*	*GGPS1*
RPS28	*NUP54*	*GGT1*
RPS29	*NUPL1*	*GGT5*
RPS3	*RPE*	*GK2*
FAU	*TPI1*	*GBA*
RPS3A	*AGRN*	*CGA*
RPS4X	*DCXR*	*GLIPR1*
RPS4Y1	*EPM2A*	*GK*
RPS5	*KIAA1199*	*GLTP*
RPS6	*NUP205*	*GPD1*
RPS7	*NHLRC1*	*GPD2*
RPS8	*NUP93*	*PNPLA4*
RPS9	*NUP98*	*GPX1*
RPSA	*BCAN*	*GPX2*
RPLP0	*SLC45A3*	*GPX4*
RPLP1	*STAB2*	*TECR*
RPLP2	*SEH1L*	*HAO2*
RPL10	*ST3GAL6*	*HADH*
RPL10A	*NUPL2*	*ALAS1*
RPL11	*NUP85*	*HEXA*
RPL12	*NUP35*	*HEXB*
RPL13	*ADPGK*	*HMGCS2*
RPL13A	*B3GNT4*	*HMGCS1*
RPL14	*B3GNT7*	*HACL1*
RPL15	*B3GALT6*	*HRASLS5*
RPL17	*CSGALNACT1*	*HRASLS*
RPL18	*CSGALNACT2*	*HRASLS2*
RPL18A	*CHPF2*	*PLA2G16*
RPL19	*CHSY1*	*EPHX2*
RPL21	*CHPF*	*IDH1*
RPL22	*CHSY3*	*IDI1*
RPL23	*CHST1*	*IDI2*
RPL23A	*CHST2*	*FABP6*
RPL24	*CHST3*	*KPNB1*
RPL26	*CHST5*	*INSIG1*
RPL26L1	*CHST6*	*PRKACA*
RPL27	*CHST7*	*PRKACB*
RPL27A	*CHST9*	*PRKACG*
RPL28	*CHST11*	*CSNK1G2*

RPL29	*CHST12*	*CSNK2A2*
RPL3	*CHST13*	*CSNK2B*
RPL3L	*CHST14*	*GPCPD1*
RPL30	*CSPG4*	*CHKB*
RPL31	*CSPG5*	*CHKA*
RPL32	*DSEL*	*MVK*
RPL34	*DSE*	*PRKD1*
RPL35	*GLB1L*	*PRKD3*
RPL35A	*GLYCTK*	*PRKD2*
RPL36	*GPC2*	*PTGR1*
RPL37	*SLC2A14*	*LTC4S*
RPL37A	*HS6ST1*	*LCAT*
RPL38	*HS6ST2*	*SPTLC1*
RPL39	*HS6ST3*	*SPTLC2*
RPL4	*HGSNAT*	*ACSL1*
UBA52	*HPSE2*	*ACSL3*
RPL41	*HPSE*	*ACSL4*
RPL36A	*HS2ST1*	*ACSL5*
RPL5	*HYAL1*	*ACSL6*
RPL6	*HYAL3*	*LDLR*
RPL7	*LYVE1*	*VLDLR*
RPL7A	*NDST3*	*LIPA*
RPL8	*NDST4*	*PNLIPRP1*
RPL9	*NUP188*	*LIPG*
AUH	*HS3ST1*	*LIPF*
PSMC1	*HS3ST2*	*LIPC*
PSMC4	*HS3ST3A1*	*LPL*
PSMC2	*HS3ST3B1*	*PNLIP*
PSMC3	*HS3ST4*	*LIPE*
PSMC6	*HS3ST5*	*LTA4H*
PSMA1	*HS3ST6*	*ALOX15*
PSMA2	*PGM2*	*ALOX5*
PSMA3	*NUP210*	*ALOX12*
PSMA4	*PPP1R3C*	*ALOX12B*
PSMA5	*RSC1A1*	*LPIN1*
PSMA6	*SLC35B2*	*LPIN2*
PSMA7	*SLC35B3*	*LPIN3*
PSMB1	*SLC35D2*	*LHB*
PSMB2	*SLC5A10*	*ALOX15B*
PSMB3	*CHST15*	*DMGDH*
PSMB4	*UST*	*ME1*
PSMB5	*XYLB*	*SLC25A20*
PSMB6	*XYLT1*	*DPEP1*
PSMB7	*XYLT2*	*ABCB4*
PSMB8	*G6PC2*	*MED6*
PSMB9	*G6PC3*	*STARD3NL*
PSMB10	*SLC5A9*	*MAPKAPK2*
PSMD1	*GNPDA2*	*STARD3*
PSMD2	*POM121*	*ABCC1*
PSMD3	*POM121C*	*ABCC3*
PSMD4	*SLC37A4*	*MBTPS1*
PSMD5	*PAPSS2*	*MBTPS2*
PSMD6	*GCK*	*MTM1*
PSMD7	*PPP2R5D*	*MTTP*
PSMD8	*UGP2*	*MTMR1*
PSMD9	*UBC*	*MTMR2*
PSMD10	*UBB*	*MTMR3*
PSMD11		*MTMR6*

PSMD12	*MTMR7*
PSMD13	*MUT*
PSME1	*NCOA2*
PSME2	*NEU1*
PSME3	*NEU2*
PSMF1	*NEU3*
RPL39L	*TRIB3*
SERINC2	*SCP2*
TPH2	*NPAS2*
PAOX	*NPC1*
ADO	*NPC2*
GRHPR	*NR1D1*
CCBL1	*NRF1*
ALDH18A1	*NR1H2*
RPS27L	*NSDHL*
SMOX	*SMPD2*
LARS	*SLC10A2*
AFMID	*SLC10A1*
AMDHD1	*CROT*
APIP	*OSBPL1A*
ASRGL1	*OSBPL2*
BHMT2	*OSBPL3*
C9orf41	*OSBPL5*
DHTKD1	*OSBPL6*
DUOX1	*OSBPL7*
DUOX2	*OSBPL8*
MRI1	*OSBPL9*
ENOPH1	*OSBPL10*
ETHE1	*OSBP*
GADL1	*PIK3CA*
GSR	*PIK3CB*
IDO2	*PIK3CD*
CCBL2	*PIK3CG*
ASPG	*EP300*
ADI1	*PIK3R3*
NMRAL1	*PIK3R1*
PYCR2	*PIK3R2*
PYCRL	*PLA2G1B*
PPM1K	*PLA2G3*
PSMB11	*PLA2G4A*
PSME4	*PLA2G5*
PSTK	*PLA2G6*
RPL10L	*PLA2G2A*
RPL22L1	*PLA2G2D*
RPL36AL	*PLA2G2E*
SCLY	*PLA2G2F*
SEPSECS	*PLA2G10*
CARNS1	*PLA2G12A*
LIPT2	*PHYH*
SERINC4	*PCCA*
SERINC5	*PCCB*
PAPSS2	*PCSK5*
TH	*STARD10*
TPO	*PCYT2*
TPH1	*P4HB*
ASMT	*PEMT*
DIO1	*HPGDS*
IYD	*HPGD*

GPT2	*PTGS1*
DIO2	*PTGS2*
ADC	*PTGDS*
	PI4KA
	PIP4K2A
	CDIPT
	PIK3C2B
	PIK3C2G
	AGPAT1
	AGPAT2
	AGPAT3
	AGPAT4
	AGPAT5
	PLD2
	PLIN1
	GPAM
	PLTP
	SLC25A17
	PMVK
	ACOT13
	PON1
	PON2
	PON3
	PPP1CA
	PPP1CB
	PPP1CC
	PPARA
	PPARD
	ALPI
	PCTP
	PITPNB
	PPT1
	PPT2
	CTSA
	PTDSS1
	ACOT8
	ACOT2
	PTEN
	PTGES
	PTGIS
	PTPN13
	RAB14
	RAB5A
	RELN
	RGL1
	RORA
	RXRA
	SLCO1A2
	SLCO1B1
	SLCO1B3
	SEC23A
	SEC24A
	SEC24B
	SEC24C
	SEC24D
	SRD5A1
	GM2A
	PSAP

SAR1B
SCAP
OXCT1
FHL2
SOAT1
SOAT2
SP1
SPHK1
SPHK2
MED21
SREBF2
STAR
STARD4
STARD5
STARD6
STARD7
STS
SULT2A1
MED22
SYNJ1
SYNJ2
MED24
THRAP3
MED12
MED13
TBL1XR1
TBL1X
TEAD1
TEAD2
TEAD3
TEAD4
PTGES3
TAZ
TBXAS1
ACOT11
THRSP
ACAA1
ACAT1
ACAA2
PLIN3
RARRES3
TPTE
TNFRSF21
TXNRD1
SLC25A1
VAPA
VAPB
HDLBP
SLC27A2
LPGAT1
FIG4
YAP1
ZDHHC8
CDK8
RAN
RAB4A
PLD1
LBR

MCEE
HMGCL
PNPLA3
AMN
CERK
TMEM55B
CRLS1
DDHD1
MMAA
PI4KB
INPP5J
PCSK9
EPT1
SGPL1
SH3KBP1
INPP5K
SIN3A
SIN3B
MED31
SGPP1
SGPP2
SCARB1
SUMF1
SUMF2
MED20
HSD3B7
B3GALNT1
ARNTL
BMP1
COL4A3BP
CHDH
ACOT1
ALDH3A2
MCAT
HMGCR
INPP5E
MGLL
MYLIP
PLA2G4C
PIP4K2B
PLEKHA1
PLEKHA2
PLEKHA3
PLEKHA4
PLEKHA5
PLEKHA6
PPARGC1A
PTDSS2
ACOT4
RUFY1
SARDH
OLAH
SGMS1
SGMS2
SREBF1
ACAT2
BAAT
THEM4

LDLRAP1
APOA5
PIK3C3
PIK3R4
CH25H
SLC27A5
TGS1
CSNK2A1
CYP4F22
MED25
MED14
MED30
MED4
AACS
ABHD3
ABHD4
ABHD5
ACBD4
ACBD5
ACBD6
ACBD7
ACSBG1
ACSBG2
ACAD10
ACAD11
ACOT6
ACOXL
ACSF2
ACSF3
ACSM3
ACSS3
NCEH1
AGK
ANKRD1
ARSG
ARSH
ARSI
ARSJ
ARSK
ARV1
ACER2
ASAH2
ACER1
AWAT1
AWAT2
BDH2
CARM1
CBR4
CDK19
CEPT1
CHD9
CHPT1
COQ2
COQ5
COQ9
CREB3L3
SLC44A1
SLC44A2

SLC44A3
SLC44A4
SLC44A5
CUBN
DDHD2
DECR2
DEGS1
DEGS2
DGAT2L6
DGAT2
HSD17B11
HSD17B12
HSD17B13
HSD17B14
PDSS2
DPEP2
DPEP3
PDSS1
ELOVL5
ELOVL6
ELOVL7
ENPP6
ENPP7
CES3
ESYT1
ESYT2
ESYT3
FAAH2
FABP9
FAR1
FAR2
FADS1
FADS2
GBA2
GDE1
GDPD1
GDPD3
GDPD5
GLB1L
GLTPD1
GPD1L
GRHL1
GPIHBP1
INPP4A
INPP4B
INSIG2
LIPH
LIPI
LIPJ
LIPK
LIPM
LIPN
PNLIPRP3
LMF1
LMF2
LSR
LCLAT1
MBOAT1

MBOAT2
LPCAT3
MBOAT7
MED13L
MED10
MED11
MED18
MED19
MED28
MED29
MED9
MFSD2A
MOGAT1
MOGAT2
MOGAT3
MTF1
MTMR4
MTMR14
NPC1L1
SMPD3
SMPD4
NUDT19
NUDT7
ORMDL1
ORMDL2
ORMDL3
PIK3C2A
PI4K2A
PI4K2B
PLA2G4D
PLA2G4E
PLA2G4F
LPCAT1
LPCAT2
PECR
PTGES2
PHOSPHO1
PIK3R5
PIK3R6
PIP4K2C
PIP5K1A
PIP5K1B
PIP5K1C
PITPNM1
PITPNM2
PITPNM3
PLEKHA8
PLA1A
PLA2R1
PLB1
PLBD1
AGPAT6
LPCAT4
AGPAT9
PLD3
PLD4
PNPLA2
PNPLA5

PNPLA6
PNPLA7
PNPLA8
GPAT2
ACP6
PPM1L
PPARGC1B
PTPLAD1
PTPLAD2
PTPMT1
PEX11A
SLC27A1
SLC27A3
TECRL
SRD5A3
SACM1L
SCD5
INPP5D
INPPL1
SMARCD3
TNFAIP8
THEM5
TMEM86B
TNFAIP8L1
TNFAIP8L2
TPTE2
FITM1
VAC14
WWTR1
PLD6
PTGR2
CHAT
C10orf129
AHRR
FAM120B
HMGCLL1
PGS1
INPP5F
TNFAIP8L3
NCOA6
CYP4A22
FABP12
PTPLA
PTPLB
HDAC3
NCOR1
MED26
CLTA
NFYA
PCSK6
PPARG
ABCG1
HSD17B7
CLTC
OCRL
ACACA
NR1H3
NFYC

MED1
NCOA1
MED27
CYP2U1
TIAM2
MED8
NR1H4
MED15
MED23
MED16
NCOR2
NCOA3
PISD
NEU4
AKR1B15

Table S3. Enrichment of metabolic pathways (pre-aGvHD vs. non-aGvHD).

**Metabolism**	**logFC**	***P*.Value**	**adj.*P*.Val**
Carbohydrate	0.084302	0.000303	0.002123
TCA cycle	0.184476	0.000724	0.002534
Amino acid	0.095083	0.005718	0.013341
Nucleotide	0.083281	0.011882	0.020794
Energy	0.057725	0.050424	0.070593
Lipid	0.009884	0.350773	0.409235
Vitamin & cofactor	0.016199	0.608394	0.608394

Table S4. Differentially expressed glycolytic genes between T cells from pre-aGvHD group and those from non-aGvHD group.

	**DEGs**	**logFC**	***P*.Value**	**adj.*P*.Val**
Upregulated	PFKP	0.265544	4.00E-07	9.59E-06
	SOD1	0.237688	6.66E-05	0.0008
	ENO3	0.239313	0.000359	0.002157
	GAPDH	0.176597	0.001011	0.004045
	STMN1	0.221446	0.001249	0.004281
	TXN	0.327283	0.002263	0.00679
	PPP2CA	0.219066	0.003088	0.008235
	PFKM	0.095339	0.004119	0.009886
	ALDOA	0.164654	0.005226	0.011402
	NT5E	0.142791	0.010599	0.021197
	PSMC4	0.194886	0.013476	0.021614
	TPI1	0.160069	0.025617	0.03586
	PC	0.160032	0.028231	0.03586
	PRKACB	0.174825	0.032698	0.039237
	ALDOB	0.148049	0.045163	0.047235
	KIF2A	0.186761	0.045848	0.047235
	PHKA2	0.082219	0.047223	0.047235
	PGM2	0.266927	0.047235	0.047235
Downregulated	B3GAT3	-0.232478787	0.000308901	0.002156761
	PAXIP1	-0.277298883	0.000540432	0.002594074
	ZNF292	-0.274517741	0.013090871	0.021614244
	ABCB6	-0.106362052	0.013508903	0.021614244
	ECD	-0.229031613	0.025125891	0.035860104
	AGRN	-0.033428452	0.028389249	0.035860104

DEGs: differentially expressed genes

Table S5. Enrichment of hallmark and TCR signaling pathways (pre-aGvHD vs. non-aGvHD).

**Signaling pathways**	**logFC**	***P*.Value**	**adj.*P*.Val**
mTORC1 signaling	0.137209087	0.001820231	0.012667985
Hedgehog signaling	0.127419678	0.002640287	0.012667985
Myc targets	0.183389787	0.002714568	0.012667985
Wnt/β-catenin signaling	0.086762945	0.035240646	0.123342262
TCR signaling pathway	0.06961957	0.049800311	0.139440871

Table S6. TFs predicted by "UCSC_TFBS".

TFs	Count	Ratio (%)	*P*.Value	Fold Enrichment	Bonferroni	Benjamini	FDR
MZF1	331	48.25073	5.29E-06	1.207865	9.37E-04	6.98E-04	2.92E-04
E2F	383	55.8309	7.88E-06	1.172615	0.001394	6.98E-04	2.92E-04
RFX1	405	59.0379	1.58E-05	1.154485	0.00279	7.66E-04	3.20E-04
MYCMAX	416	60.6414	1.73E-05	1.148356	0.003059	7.66E-04	3.20E-04
TCF11	338	49.27114	6.48E-05	1.172985	0.011407	0.001846	7.72E-04
STAT3	315	45.91837	8.59E-05	1.182126	0.015082	0.001846	7.72E-04
HMX1	305	44.46064	8.97E-05	1.187415	0.015747	0.001846	7.72E-04
NFY	325	47.37609	9.30E-05	1.175385	0.016333	0.001846	7.72E-04
HNF4	320	46.64723	9.39E-05	1.178051	0.016479	0.001846	7.72E-04
ATF6	306	44.60641	1.41E-04	1.18027	0.024721	0.002503	0.001046
YY1	454	66.18076	1.58E-04	1.112129	0.027624	0.002546	0.001065
NFKAPPAB50	159	23.17784	2.33E-04	1.301378	0.040393	0.003436	0.001436
IK2	206	30.02915	2.74E-04	1.241393	0.047381	0.003733	0.001561

Figure S1

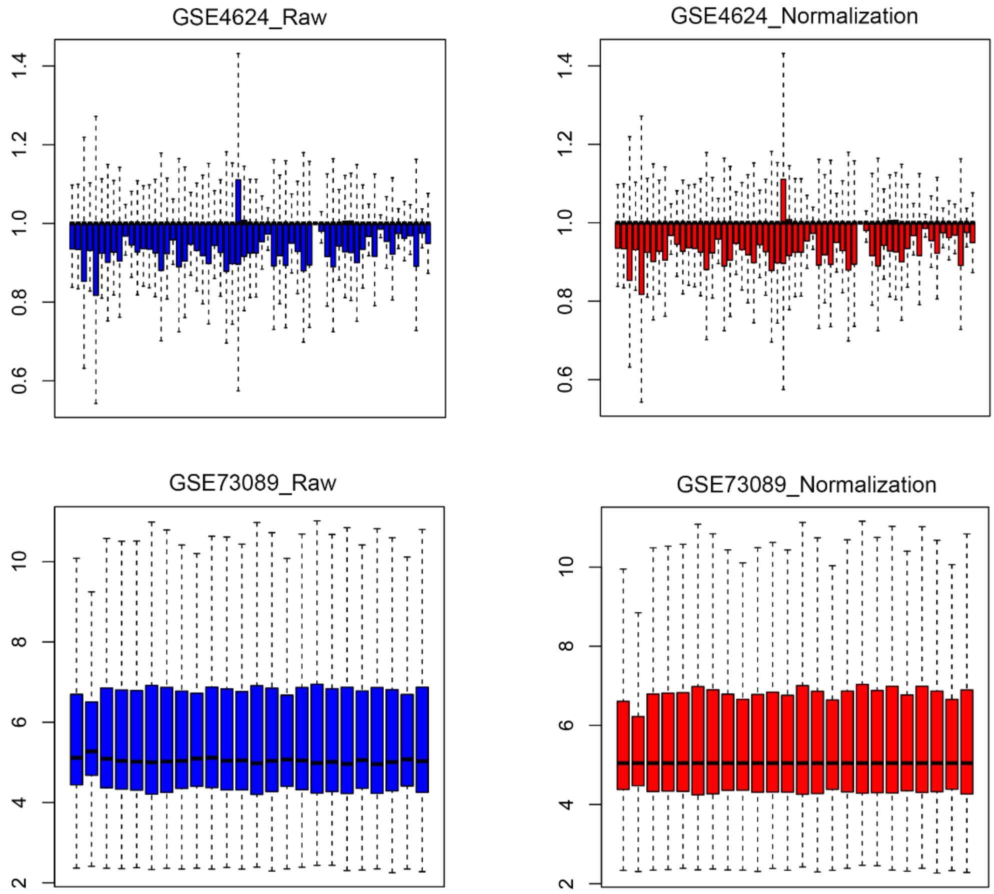

Figure S1. Normalization of GSE4624 and GSE73809 raw data. Blue represents raw data and red represents normalized data.

Figure S2

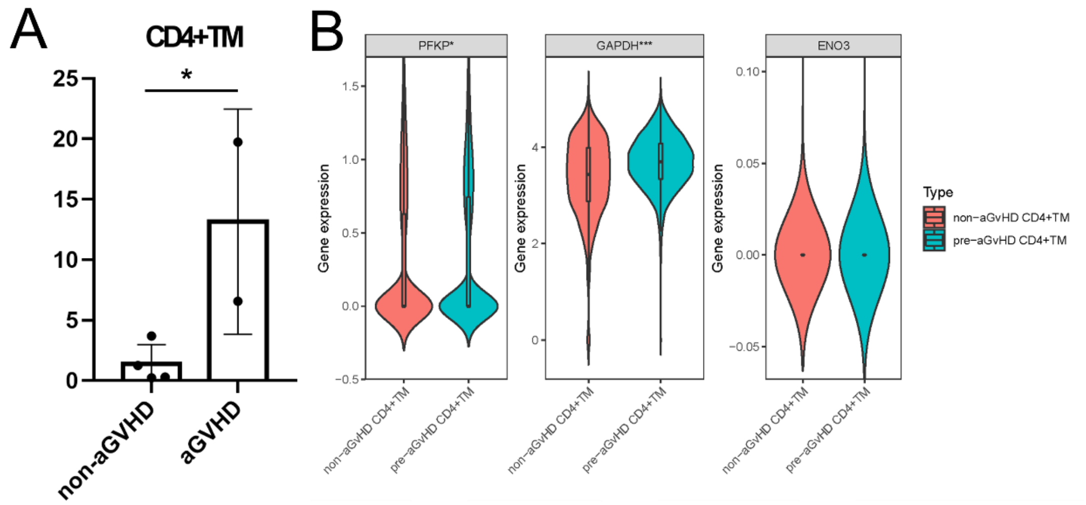

Figure S2. Proportion of CD4⁺ memory T cells and expression of glycolytic genes in aGvHD patients. (A) Proportion of CD4⁺ memory T cells in aGvHD patients (n=2) and non-aGvHD patients (n=4). (B) Violin plots showing expression of *PFKP*, *GAPDH* and *ENO3* in non-aGvHD CD4 memory T cells (n=1310) and pre-aGvHD CD4 memory T cells (n=2485) by scRNA sequencing. Black dots denote mean values; widths denote cell densities. TM, memory T cells. **P*<0.05, ***P*<0.05, ****P*<0.001.

Figure S3

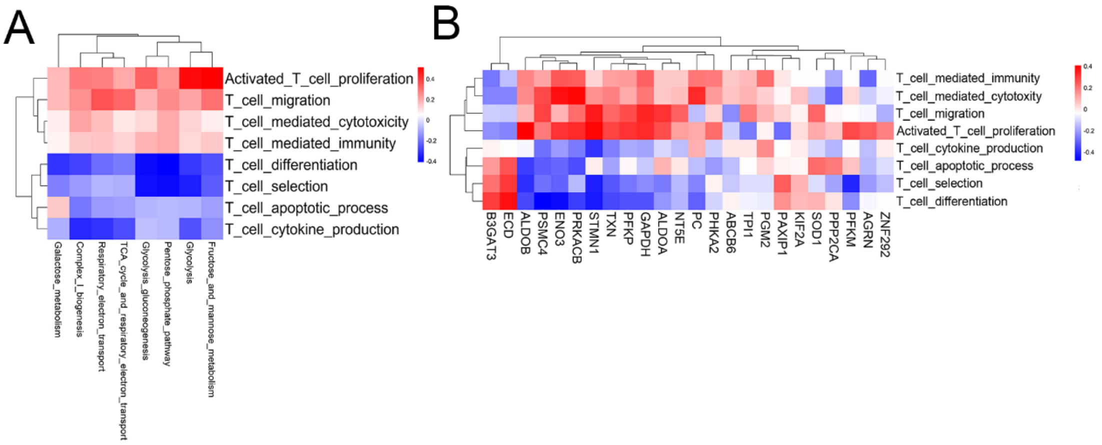

Figure S3. T-cell functional phenotypes associated with glycolysis-related pathways and glycolytic genes. (A) Heat map of the Pearson correlation coefficients between glycolysis-related metabolic signatures and T-cell functional phenotypes. (B) Heat map of the Pearson correlation coefficients between glycolytic genes and T-cell functional phenotypes. The T-cell functional gene-sets are based on GO biological process. Red, positive correlation; blue, negative correlation.

Figure S4

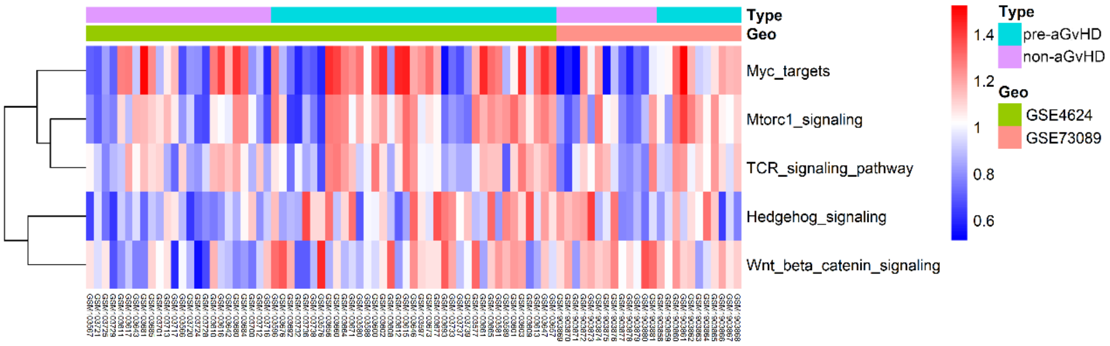

Figure S4. Hallmark and TCR signaling enrichment based on pathway gene expression. (A) Heatmap shows significant enrichment of hallmark and TCR signaling signatures across each sample. The right longitudinal axis indicates the names of pathways. The left longitudinal axis represents clustering of pathways. Red denotes high activity and blue denotes low activity of signaling pathways respectively. The hallmark signaling and TCR signaling gene sets are based on MSigDB.

Figure S5

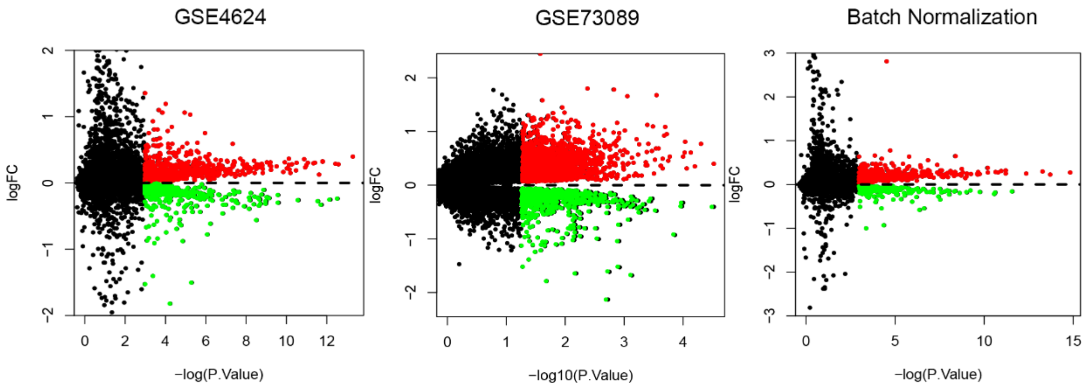

Figure S5. Volcano plots of differentially expressed genes. The red dots represent the upregulated genes; the green dots represent the downregulated genes; the black spots represent genes with no significant difference in expression. Differentially expressed genes with $P < 0.05$ and |log FC| > 1 were considered significant. FC, fold change.

Figure S6

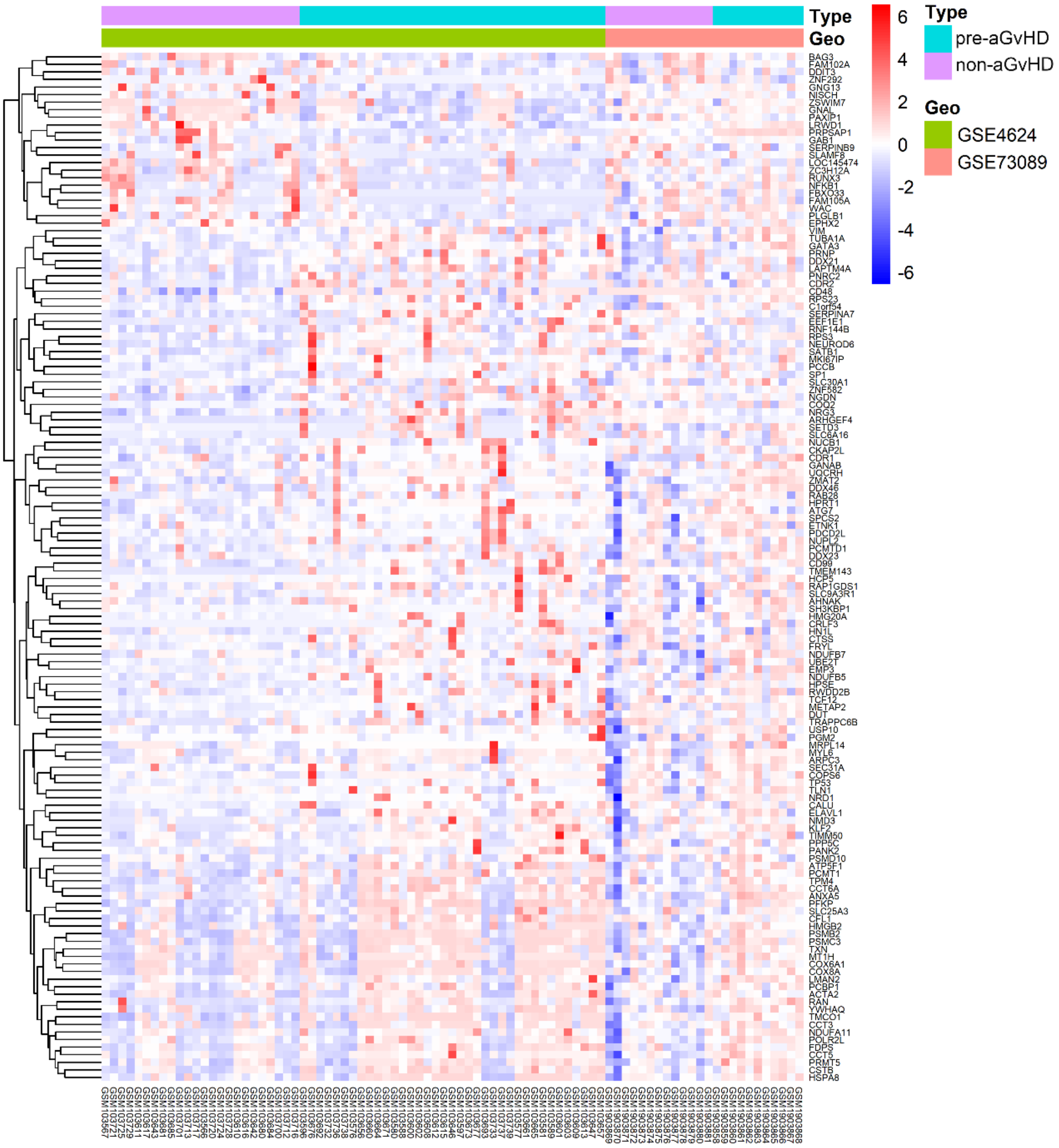

Figure S6. Heatmap and hierarchical clustering of differentially expressed genes between T cells from allo-HSCT patients who further developed aGvHD and without aGvHD in GEO database. The right longitudinal axis indicates names of differentially expressed genes. The left longitudinal axis represents clustering information. The red color represents the upregulated genes; the blue color represents the downregulated genes; the white color represents genes without change. Significance was defined in Figure 2A, above.

Figure S7

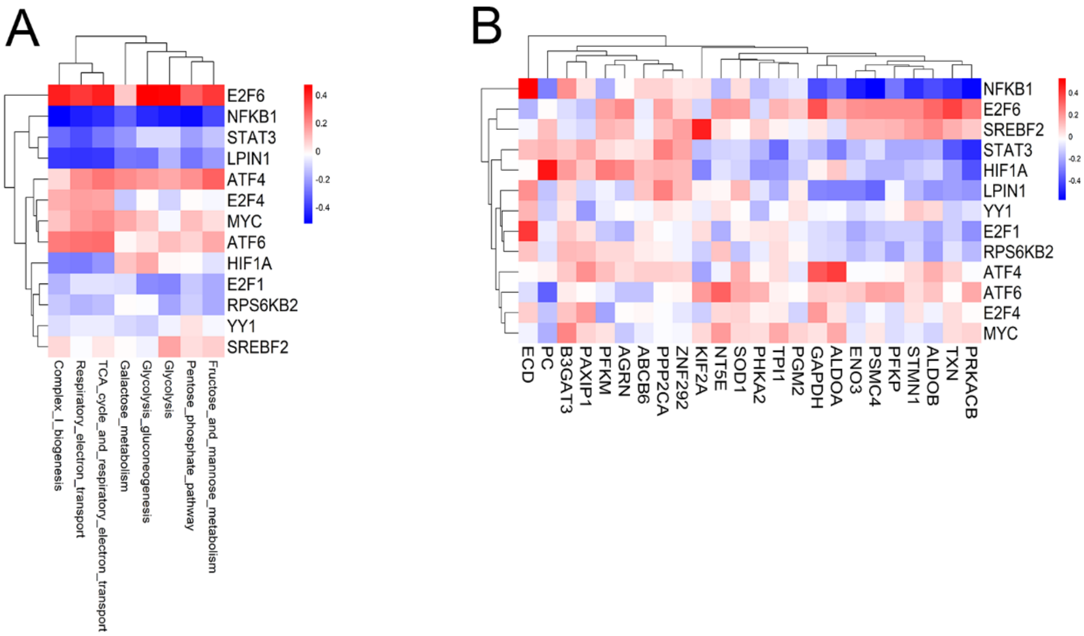

Figure S7. TFs associated with glycolysis-related pathways and glycolytic genes. (A) Heat map of the Pearson correlation coefficients between glycolysis-related metabolic signatures and potential TFs. (B) Heat map of the Pearson correlation coefficients between glycolytic genes and potential TFs.

Figure S8

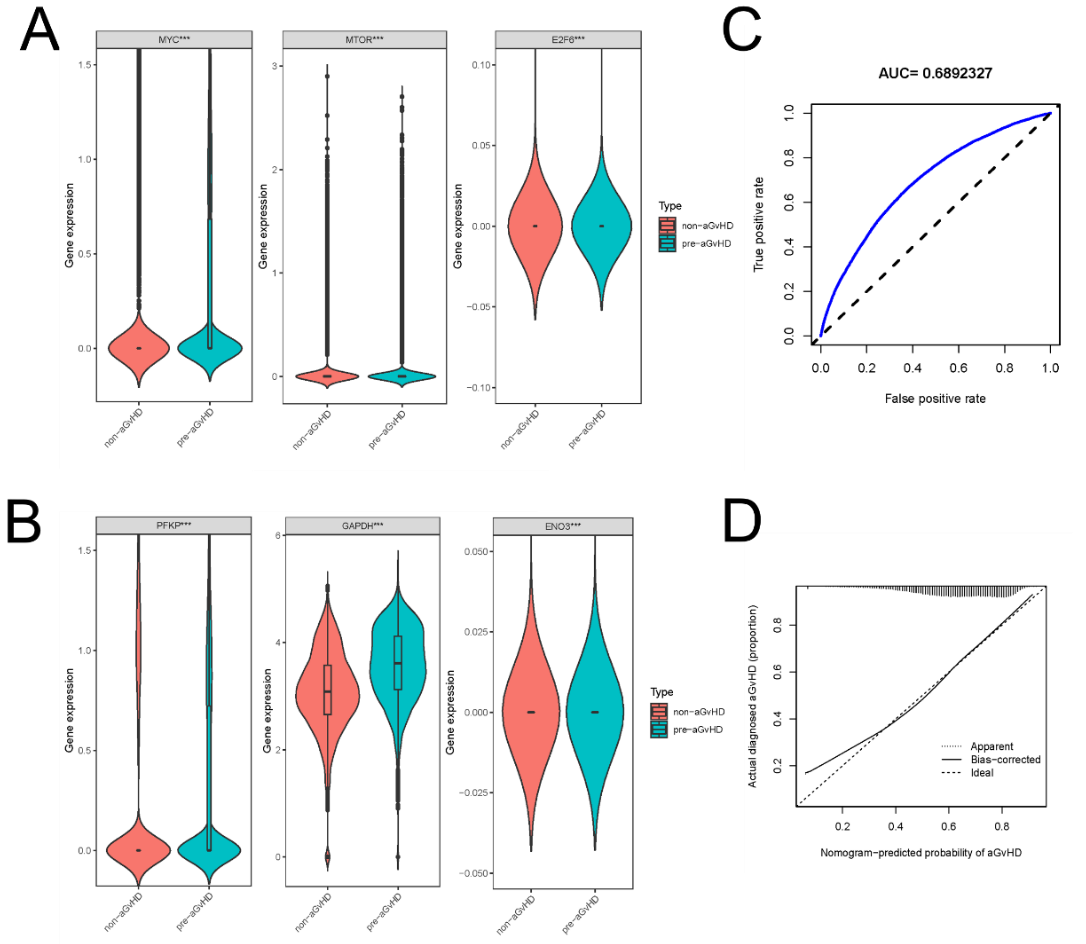

Figure S8. Validation of aGvHD risk model. (A) Violin plots showing expression of *MYC*, *MTOR* and *E2F6* in non-aGvHD T cells (n=18286) and pre-aGvHD T cells (n=30432) by scRNA-sequencing. (B) Violin plots showing expression of *PFKP*, *GAPDH* and *ENO3* in non-aGvHD T cells (n=18286) and pre-aGvHD T cells (n=30432) by scRNA-sequencing. Black dots denote mean values; widths denote cell densities. (C) ROC curve shows predictive value of the aGvHD risk model in our patient cohort. (D) Calibration curves of the aGvHD risk model in our patient cohort.

Figure S9

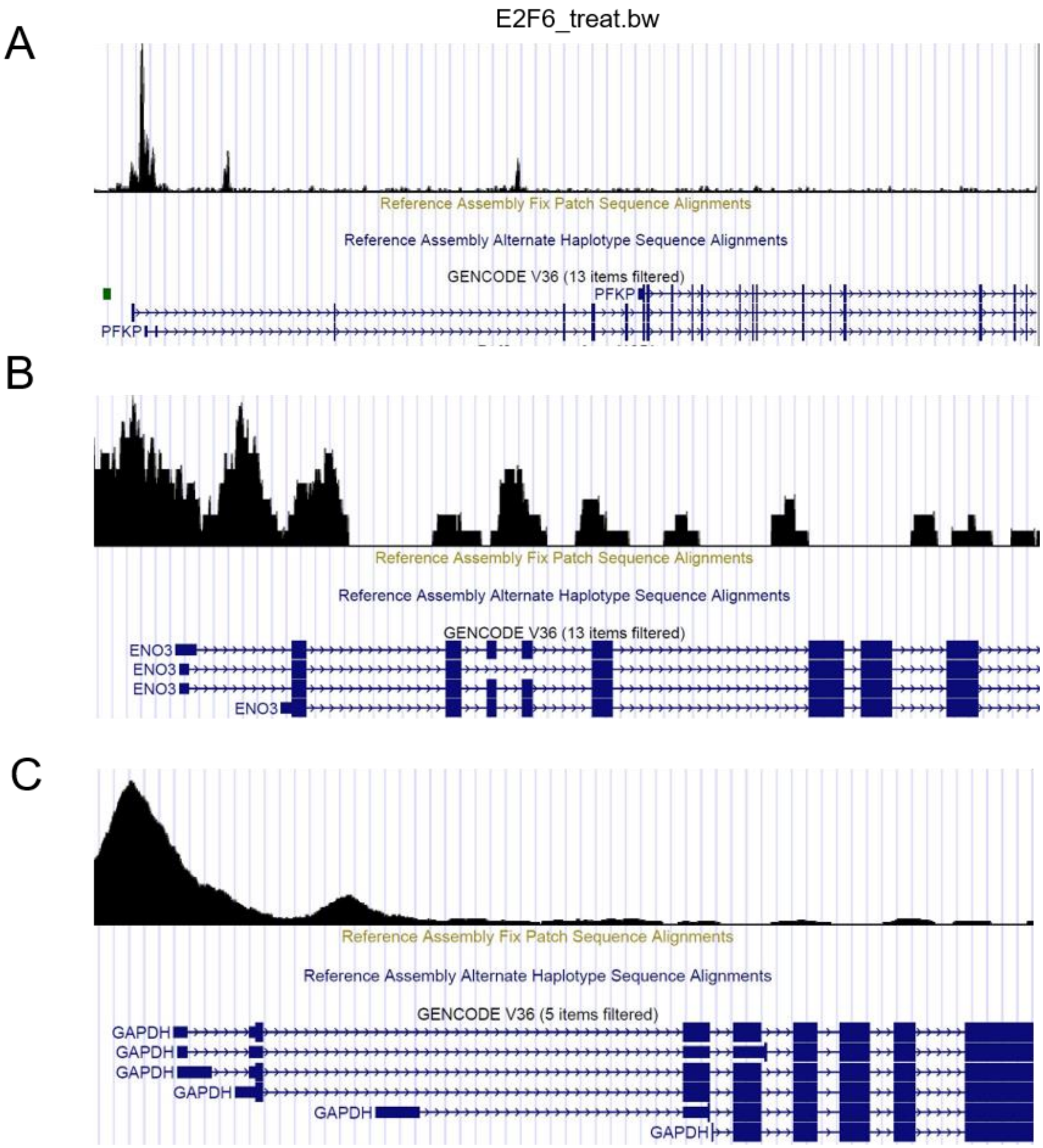

Figure S9. ChIP-seq data predicted E2F6 as TF regulating the expression of *PFKP*, *ENO3* and *GAPDH*. Using hematopoietic cell-specific ChIP-seq data collected in Cistrome data browser, E2F6 was predicted to bind the promoter region of *PFKP* (A), *ENO3* (B) and *GAPDH* (C).

Figure S10

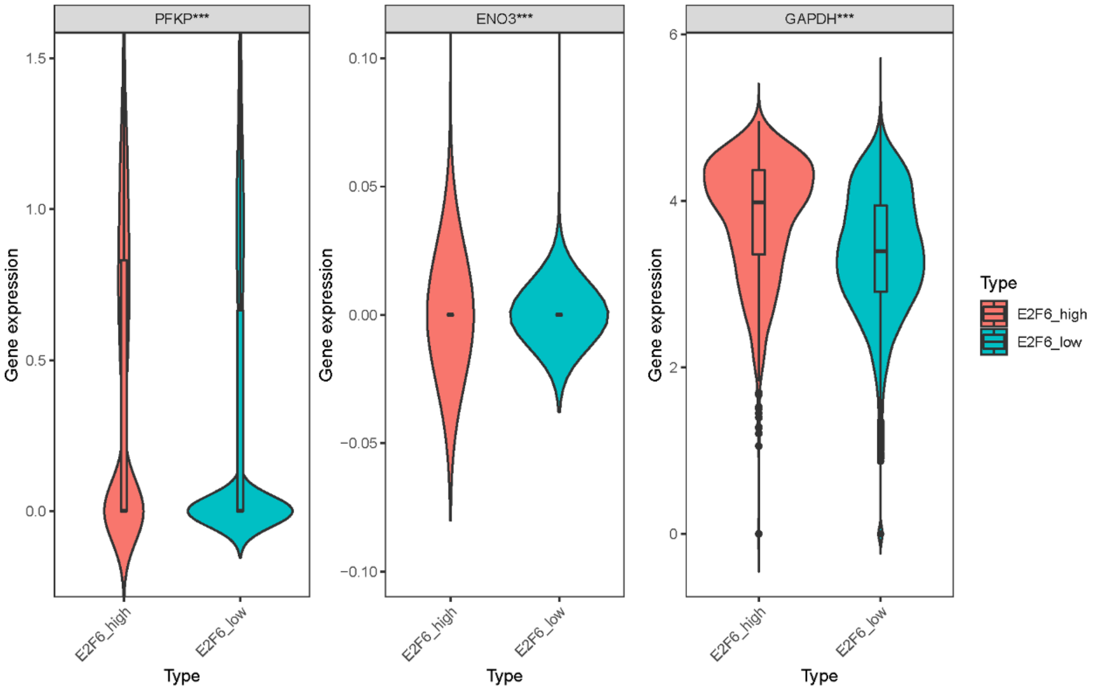

Figure S10. Expressions of *PFKP, ENO3 and GAPDH* in correlation with *E2F6* expression. Violin plots comparing the expressions of *PFKP, ENO3* and *GAPDH* in alloreactive T cells with lower *E2F6* expression (n=47219) and alloreactive T cells with upper *E2F6* expression (n=1499) by scRNA-sequencing. Mean value defined upper expression or lower expression. **P*<0.05, ***P*<0.05, ****P*<0.001.
